# Single-cell analysis reveals fibroblast heterogeneity and myeloid-derived adipocyte progenitors in murine skin wounds

**DOI:** 10.1038/s41467-018-08247-x

**Published:** 2019-02-08

**Authors:** Christian F. Guerrero-Juarez, Priya H. Dedhia, Suoqin Jin, Rolando Ruiz-Vega, Dennis Ma, Yuchen Liu, Kosuke Yamaga, Olga Shestova, Denise L. Gay, Zaixin Yang, Kai Kessenbrock, Qing Nie, Warren S. Pear, George Cotsarelis, Maksim V. Plikus

**Affiliations:** 1 Department of Developmental and Cell Biology, University of California, Irvine, Irvine, CA 92697 USA; 2 Sue and Bill Gross Stem Cell Research Center, University of California, Irvine, Irvine, CA 92697 USA; 3 Center for Complex Biological Systems, University of California, Irvine, Irvine, CA 92697 USA; 4 Department of Pathology and Laboratory Medicine, Abramson Family Cancer Research Institute, Institute for Medicine and Engineering, University of Pennsylvania, Philadelphia, PA 19104 USA; 5 Department of Mathematics, University of California, Irvine, Irvine, CA 92697 USA; 6 Department of Biological Chemistry, University of California, Irvine, Irvine, CA 92697 USA; 7 CEA/INSERM Inserm_U967, 92265 Fontenay-aux-Roses cedex, France; 8 Department of Dermatology, Kligman Laboratories, Perelman School of Medicine at the University of Pennsylvania, Philadelphia, PA 19104 USA

## Abstract

During wound healing in adult mouse skin, hair follicles and then adipocytes regenerate. Adipocytes regenerate from myofibroblasts, a specialized contractile wound fibroblast. Here we study wound fibroblast diversity using single-cell RNA-sequencing. On analysis, wound fibroblasts group into twelve clusters. Pseudotime and RNA velocity analyses reveal that some clusters likely represent consecutive differentiation states toward a contractile phenotype, while others appear to represent distinct fibroblast lineages. One subset of fibroblasts expresses hematopoietic markers, suggesting their myeloid origin. We validate this finding using single-cell western blot and single-cell RNA-sequencing on genetically labeled myofibroblasts. Using bone marrow transplantation and Cre recombinase-based lineage tracing experiments, we rule out cell fusion events and confirm that hematopoietic lineage cells give rise to a subset of myofibroblasts and rare regenerated adipocytes. In conclusion, our study reveals that wounding induces a high degree of heterogeneity among fibroblasts and recruits highly plastic myeloid cells that contribute to adipocyte regeneration.

## Abstract (web summary)

The diversity of fibroblasts contributing to wound healing is unclear. Here, the authors use single-cell RNA-sequencing to identify heterogeneity among murine fibroblasts in the wound and find that recruited myeloid cells contribute to adipocyte regeneration during healing.

## Introduction

Skin forms the outermost layer of the body, and principally consists of a stratified epidermis residing on top of a collagen-rich dermis. While epidermis endows skin with its barrier function, dermis provides mechanical strength and houses numerous epidermal appendages, principally hair follicles and sweat glands. Hair follicles are complex epithelial–mesenchymal mini-organs that are rich in stem cells and regenerate cyclically. When fully grown, hair follicles span the entire dermis and part of the dermal white adipose tissue (dWAT), where they engage in signaling crosstalk. As a result of this crosstalk, hair follicles induce adipocyte progenitor proliferation and adipocyte hypertrophy^1^. Reciprocally, dWAT modulates hair stem cell quiescence and activation^2,3^.

Upon significant injury, such as full-thickness excisional wounding, skin undergoes repair. While small wounds, <1 cm^2^, typically repair by forming scar devoid of epidermal appendages and fat, large wounds, larger than 1 cm^2^, can regenerate de novo hair follicles^4^ and adipocytes in their center^5^. Large wounds in mice heal primarily by contraction, while the uncontracted portion closes by re-epithelialization and forms new connective tissue, rich in fibroblasts. In our model, wounds close in two weeks, and then new hair follicles regenerate in the central region by week three^4,6^, followed by new adipocytes during the fourth week^5^. The process of de novo hair follicle regeneration, termed wound-induced hair neogenesis (WIHN), involves reactivation of embryonic hair development programs^4^. Similarly, the process of de novo fat regeneration involves reactivation of an embryonic adipose lineage formation program^5^ (Supplementary Figure 1). It remains unclear why regeneration is limited to the wound center. Beyond laboratory mice^4,6,7^, WIHN is observed in rodents from the genus *Acomys*^8^ and in rabbits^9^. WIHN also likely occurs in sheep^10^, but not in laboratory rats^11^. While typically wounds in humans heal without regeneration, de novo hair follicles in facial skin wounds have been reported^12^.

Over the last decade, many signaling pathways necessary for WIHN have been partially elucidated. Activation of canonical WNT signaling is necessary for WIHN^4,6,13^, and both epidermal^13^ and dermal wound cells secrete and respond to WNT ligands at distinct phases^6^. Production of WNT ligands by dermal wound cells is initiated by FGF9, secreted by γδ T cells^6^. Activation of Hedgehog signaling downstream of WNT is necessary for dermal papilla (DP) fate specification by wound fibroblasts and for successful WIHN^14^. Macrophages also promote WIHN by secreting TNFα, which, in turn, activates p-AKT/p-β-catenin signaling^15^. Activation of TLR3 signaling by double-stranded RNA augments IL6 production and STAT3 activation, both of which positively impact WIHN efficiency^7^. In contrast, prostaglandin PDG2 inhibits hair follicle neogenesis^16^. Furthermore, WIHN is modulated by several transcriptional regulators, including homeobox factor MSX2^17^, zinc finger protein CXXC5^18^, and RNA-binding protein MSI2^19^. De novo fat regeneration is driven by BMP ligands produced by neogenic hair follicles^5^. Responding to BMP signals, wound myofibroblasts activate ZFP423, a transcriptional regulator that drives adipogenic lineage commitment. Hair follicles are critical for fat neogenesis and no adipocytes regenerate in hairless wounds^5^.

Less is known about the lineage origin of cells during WIHN. Fate mapping experiments by Ito, Yang^4^ showed that progeny of the pre-existing KRT15-positive hair follicle bulge stem cells do not give rise to de novo hair follicles. Instead, progeny of non-bulge LGR6-positive^20^ and LGR5-positive epithelial stem cells^15^ can contribute toward neogenic hair follicles. Little is known about the cells that regenerate DP, the principal mesenchymal component of hair follicles. Lineage studies suggest that myofibroblasts are the source for neogenic DPs^5,14^. Tracing experiments on CD133-positive DP cells of preexisting hair follicles indicate that they do not mobilize upon wounding^21^. Other fate mapping studies suggest that multiple skin fibroblast lineages mobilize to repair small wounds^22–24^. Using lineage tracing with *En1* (*Engrailed 1*)*-Cre*, Rinkevich, Walmsley^23^ identified *En1*-positive and *En1*-negative dermal fibroblasts. After wounding, *En1*-positive fibroblasts become major contributors toward wound repair, and genetically depleting them leads to scar reduction. The contribution of *En1*-positive fibroblasts toward wound repair was confirmed by Shook, Wasko^24^. Driskell, Lichtenberger^22^ showed that dermal fibroblasts contribute to wound repair in two waves. Progeny of lower dermal fibroblasts contribute early, while progeny of upper fibroblasts migrate into the wound later. Contribution from distinct dermal fibroblasts toward regenerating DPs in the WIHN model requires further investigation, yet the lack of clear lineage master-regulators complicates functional validation of DP fate mapping.

Unlike DPs, fat tissue with its well-established master-regulators, including ZFP423, CEBPs, and PPARγ, provides a tractable model system for studying de novo regeneration. Recently, we showed that de novo adipocytes regenerate from *Sm22* (aka *Tagln*) and *Sma* (aka *Acta2*) positive myofibroblasts^5^. Myofibroblast-specific ablation of *Pparγ* or BMP receptor 1a *(Bmpr1a)* largely prevented adipocyte regeneration in otherwise hair-bearing wounds. However, the degree of wound myofibroblast heterogeneity and their competency for adipogenic reprogramming remains unclear. The advent of single-cell RNA-sequencing (scRNA-seq) enables profiling of cellular heterogeneity in tissues with poorly characterized cell types. In this study, using a scRNA-seq approach, we identify and characterize multiple distinct fibroblast populations in regenerating mouse wounds. We show that major populations co-exist in wounds across the time course of regeneration. Furthermore, we identify bone marrow-derived adipocytes and a rare subset of wound fibroblasts with myeloid characteristics that undergo fat regeneration.

## Results

### Single-cell analysis reveals heterogeneity in large wounds

**Fig. 1:**
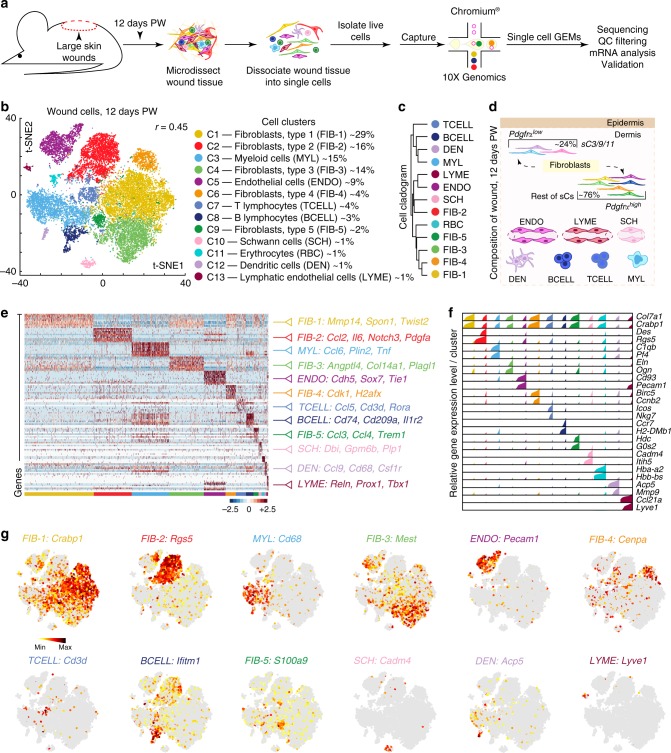
scRNA-seq analysis reveals cellular heterogeneity in day 12 wounds. **a** Schematic of cell isolation, cell processing, capture by droplet-based device, sequencing, and downstream analysis. **b** t-SNE plot revealed cellular heterogeneity with 13 distinct clusters of cells identified and color-coded. General identity of each cell cluster is defined on the right. Parameter *r* refers to Seurat’s FindClusters function and determined clustering resolution. **c** Unsupervised hierarchical clustering of average gene signatures showing relatedness of cell clusters (correlation distance metric, average linkage). **d** Wound schematic showing cellular repertoire. Different cell types, as identified on scRNA-seq, are color-coded to match colors on **b**. **e** Heatmap of differentially expressed genes. For each cluster the top 10 genes and their relative expression levels in all sequenced wound cells are shown. Selected genes for each cluster are color-coded and shown on the right. **f** Relative expression of selected cluster-specific genes shown as high-density bar charts. Bar height corresponds to gene’s relative expression level in each cell and ordering is performed from low to high expressing cells. Two genes are shown per cluster and clusters are color-coded to match the colors in **b**. **g** Feature plots of expression distribution for selected cluster-specific genes. Expression levels for each cell are color-coded and overlaid onto t-SNE plot. Cells with the highest expression level are colored black. PW: post-wounding, GEM: gel bead-in-emulsion, QC: quality control

We performed scRNA-seq on unsorted cells from wound dermis 12 days post-wounding (PW) (Fig. 1a). This time point coincides with completion of wound re-epithelialization and strong SMA expression^5^. Approximately 21,819 sequenced cells met quality control metrics (Supplementary Figure 2) and were analyzed. Unsupervised clustering using the Seurat package^25^ identified 13 cell clusters (Fig. 1b, left). Using the differentially expressed gene signatures, we attributed clusters to their putative identities (Fig. 1b, right) and hierarchical similarities (Fig. 1c; Supplementary Figure 3a). Figure 1d provides a summary diagram of identified cell types. Figure 1e–g show selected differentially expressed genes in the form of a heatmap (Fig. 1e), bar charts (Fig. 1f), and feature plots (Fig. 1g). Several clusters contained immune cells. The most abundant of them, representing ~16% of all cells, was cluster C3. It was enriched for myeloid markers, including *C1qb, Cd14, Cd68, Lyz2, Mafb*, and *Pf4* (Supplementary Figure 3b; Supplementary Data 1). Cluster C7 cells were classified as T lymphocytes (~4%) and they expressed *Cd3d, Cd81, Icos, Maf, Nkg7*, and *Thy1* (Supplementary Figure 4). Cluster C8 cells were identified as B lymphocytes (~3%) and C12 as dendritic cells (~1%). Two other distinct cell clusters were C5 (~9%) and C13 (~1%). Cluster C5 cells were enriched for endothelial markers *Cav1, Cd34, Cd93, Ly6e, Ly6c1*, and *Pecam1*, while cluster C13 cells—for lymphatic endothelial markers *Ccl21a, Lyve1, Pdpn*, and *Prox1*. Cluster C10 cells (~1%) were classified as Schwann cells based on expression of *Cadm4, Dbi, Gpm6b, Itih5*, and *Plp1*. The remaining five clusters—C1, C2, C4, C6 and C9, representing ~65% of all analyzed wound cells, were collectively characterized as fibroblasts. They were highly enriched for collagens, including *Col1a1, Col3a1, Col5a1, Col12a1*, and extracellular matrix protein genes, including *Dcn* (*Decorin*) and *Fbln2* (*Fibulin 2*) (Supplementary Figure 3b; Supplementary Data 1). Many of these cells expressed high levels of contractile myofibroblast factors: *Cald1, Myl9, Tagln*, and *Acta2* (Supplementary Figure 3b).

### Wound fibroblasts subcluster into distinct cell populations

**Fig. 2:**
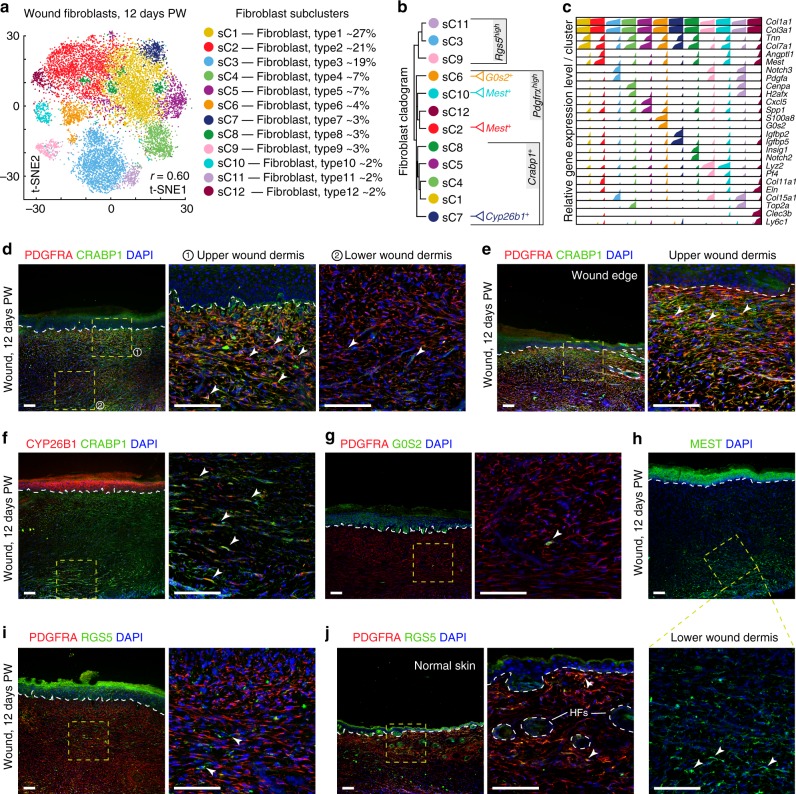
Subclustering of wound fibroblasts reveals cellular heterogeneity. **a** Subclustering of wound fibroblasts (cells from clusters C1, C2, C4, C6, and C9 shown in Fig. 1) further identified 12 distinct subtypes. Color-coded t-SNE plot is shown and each fibroblast subcluster (sC1 through sC12) is defined on the right. **b** Unsupervised hierarchical clustering showing relatedness of wound fibroblast subclusters. Immunostaining markers specific to individual fibroblast subclusters or groups of subclusters are listed on the right. **c** Relative expression of selected subcluster-specific genes shown as high-density bar charts. Bar charts are generated analogous to these in Fig. 1f. Two genes are shown per subcluster and subclusters are color-coded to match the colors in **a**. **d–j** Staining of day 12 wounds for selected markers. **d**, **e** Co-staining for PDGFRA (red) and CRABP1 (green) identifies enriched localization of CRABP1^+^/PDGFRA^+^ double-positive fibroblasts in the upper wound dermis (arrowheads on inset 1 on **d** and inset on **e**). Lower wound dermis contains large numbers of PDGFRA^+^ single-positive fibroblasts (inset 2 on **d**). **f** Co-staining for CRABP1 (green) and CYP26B1 (red) identifies occasional CYP26B1^+^/CRABP1^+^ double-positive fibroblasts (arrowheads on inset). **g** Co-staining for PDGFRA (red) and G0S2 (green) identifies seldom G0S2^+^/PDGFRA^+^ double-positive fibroblasts (arrowhead on inset). **h** Numerous MEST^+^ cells (green) are present in the wound and primarily localize in the lower wound dermis (arrowheads on inset). **i** Co-staining for PDGFRA (red) and RGS5 (green) identifies RGS5^+^ single-positive fibroblasts present throughout wound dermis (arrowheads on inset). **j** Normal, unwounded skin contains both RGS5^+^ single-positive and RGS5^+^/PDGFRA^+^ double-positive fibroblasts, especially in the upper dermis (arrowheads on inset). Hair follicles are marked. Immunostaining images shown in this figure are representative of the marker staining patterns observed in three or more independently stained samples. PW: post-wounding, HF: hair follicle. Size bars: **d**–**j**—100 µm

Next, we performed unsupervised clustering on all wound fibroblasts and observed further heterogeneity with 12 subclusters, sC1 through sC12 (Fig. 2a, b; Supplementary Figure 5), each containing unique marker gene profiles (Fig. 2c; Supplementary Figure 6; Supplementary Data 2). Considering that transcription factors (TFs) commonly regulate cellular characteristics, we examined their expression patterns. All fibroblasts shared the following 20 TFs: *Cebpb, Egr1, Fosb, Fosl2, Hif1a, Klf2, Klf4, Klf6, Klf9, Nfat5, Nfatc1, Nfkb1, Nr4a1, Nr4a2, Pbx1, Prrx1, Runx1, Stat3, Tcf4*, and *Zeb2* (Supplementary Figure 7a). These can be defined as a common wound fibroblast TF signature. Among these factors were Runx1^26^, Tcf4^27^, and Zeb2^28^ (Supplementary Figure 8), previously implicated in myofibroblast differentiation. The *Ebf1*^*high*^*/Id3*^*high*^*/Zeb2*^*high*^*/En1*^*low*^*/Nfix*^*low*^*/Prrx2*^*low*^*/Sox9*^*off*^ signature marked fibroblasts from sC3/9/11 subclusters (Supplementary Figure 7b, c), which are hierarchically distinct (Fig. 2b; Supplementary Figure 5). Among the remaining nine subclusters, fibroblasts in six subclusters, sC1 and sC4–sC8, had low expression of *Id2* and *Id3*. Among these six *En1*^*high*^*/Id2*^*low*^*/Id3*^*low*^ subclusters, sC4 was *Sox11*^*high*^, sC5–*Twist2*^*high*^, sC7–*Twist1*^*high*^*/Twist2*^*high*^*/Foxp1*^*low*^ and sC8–*Nfia*^*high*^ (Supplementary Figure 7b, c). Other subclusters also had their own, albeit complex TF expression signatures.

Next, we examined signaling pathway markers, receptors and ligands. Hierarchically distinct subclusters sC3/9/11 had the following receptor signature: *Mcam*^*high*^*/Pdgfrb*^*high*^*/Fgfr1*^*low*^*/Tgfbr2*^*low*^*/Tgfbr3*^*low*^*/Ncam1*^*off*^*/Pdgfra*^*low*^, and ligand signature: *Il6*^*high*^*/Pdgfa*^*high*^*/Igf1*^*low*^*/Igfbp3*^*low*^*/Mdk*^*low*^*/Dkk3*^*off*^. Nine remaining subclusters were primarily differentiated from subclusters sC3/9/11 based on high *Pdgfra* expression, while having low *Il6, Pdgfa*, *Pdgfrb* and high *Igf1, Mdk, Tgfbr2, Tgfbr3* expression (Supplementary Figure 7c). Additionally, among these nine subclusters, fibroblasts in sC2 were *Angptl1*^*high*^, sC5 –*Ccl8*^*high*^*/Cxcl5*^*high*^*/Grem1*^*high*^*/Spp1*^*high*^, sC6–*Il1b*^*high*^, sC7–*Ccl8*^*high*^*/Igfbp3*^*low*^, sC10–*Angptl1*^*high*^*/Il1b*^*high*^*/Pf4*^*high*^ and sC12–*Angptl1*^*high*^*/Fst*^*high*^ (Supplementary Figure 7b, c; Supplementary Data 2). We also profiled cell cycle state^29^. Intriguingly, sC4 and sC11 subclusters were prominently enriched for G2/M markers (Supplementary Figure 9). Considering hierarchical similarities (Fig. 2b), sC4 and sC11 likely represent actively cycling subsets of sC1 and sC3 populations, respectively.

We also spatially mapped a portion of fibroblast subclusters to day 12 wound regions using immunostaining. We chose markers based on their high, yet specific expression on scRNA-seq and their membranous or intracellular localization (Fig. 2b). Fibroblasts in clusters sC1/2/4-8/10/12 were marked based on PDGFRA expression. Among these, cells in subclusters sC1/4/5/7/8 were differentiated from sC2/6/10/12 cells based on CRABP1 expression. Intriguingly, PDGFRA^+^/CRABP1^+^ cells were enriched under the epidermis, while PDGFRA^+^/CRABP1^neg^ cells primarily localized to the lower dermis (Fig. 2d, e). Among CRABP1^+^ cells, rare sC7 cells (~3%) were also CYP26B1^+^ and showed scattered presence in the dermis (Fig. 2f). Among PDGFRA^+^/CRABP1^neg^ cells, rare sC6 cells (~4%) were G0S2^+^ and were seldom found in the lower dermis (Fig. 2g), while MEST2^+^ sC2/10 cells, which were abundant on scRNA-seq, showed broad presence throughout the lower dermis (Fig. 2h). sC3/9/11 fibroblasts were distinguished based on RGS5 expression. RGS5^+^ cells were present throughout wound dermis (Fig. 2i, j), and majority of them localized outside of the blood vessels.

Taken together, our analyses suggest that, upon completion of re-epithelialization on day 12, large wounds may contain two major fibroblast populations. One population, representing ~24% of wound fibroblasts, consists of three subclusters sC3/9/C11, which express low levels of TGFβ receptors *Tgfbr2, Tgfbr3*, and PDGF receptor *Pdgfra* and high levels of *Pdgfrb*. They also stain with RGS5 and localize throughout wound dermis. The second and more heterogeneous population, representing ~76% of wound fibroblasts, consists of nine subclusters and expresses intermediate to high levels of *Tgfbr2, Tgfbr3*, high levels of *Pdgfra*, but not *Pdgfrb*. Notably, PDGFRA signaling is an established driver of fibrosis in multiple tissues, including fat and skeletal muscle. *Pdgfra*^*high*^ cells also express higher levels of receptors for the TGFβ pathway, another well-established driver of fibrosis. *Pdgfra*^*low*^ cells are also *Pdgfrb*^*high*^, and *Pdgfrb*-expressing perivascular cells were identified as the precursors for new adipocytes in visceral fat in obesity^30^. Additionally, we identified a high level of previously unappreciated heterogeneity within both fibroblast populations. For instance, *Pdgfra*^*high*^ fibroblasts further subdivide into upper and lower dermal cells both anatomically and by gene expression.

### Analyses reveal myofibroblast differentiation trajectories

**Fig. 3:**
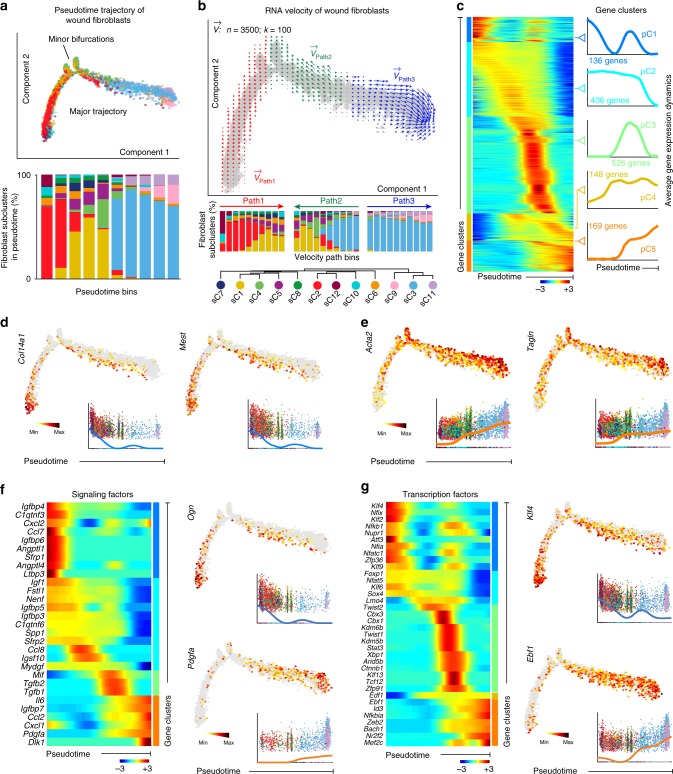
Pseudotime analyses reveal putative fibroblast differentiation trajectories. **a** Pseudotime ordering on wound fibroblasts arranged them into a major trajectory, with two minor bifurcations (top panel). Stacked bar chart shows relative abundance of fibroblasts from distinct subclusters across the pseudotime, which was divided into ten equal bins (bottom panel). *Pdgfra*^*low*^ sC3/9/11 cells primarily occupy right half of the trajectory, with the remaining part consisting of *Pdgfra*^*high*^ cells. All cells on the pseudotime and the bar chart are color-coded to match the colors in Fig. 2a. **b** RNA velocity analysis distinguished three sets of velocity vectors across the pseudotime: Path1 (red), Path2 (green) and Path3 (blue). Stacked bar charts show relative abundance of fibroblast subclusters across individual RNA velocity paths (middle panel). Bottom panel defines fibroblast subcluster colors and matches the colors in Fig. 2a. **c** scEpath analysis performed on pseudotime identified five gene clusters (pC1 through pC5) of pseudotime-dependent genes. Heatmap of the expression levels for all differentially expressed genes in all analyzed wound fibroblasts is shown on the left. Average gene expression dynamics and gene counts in all five clusters are shown on the right. **d** Feature plots of expression distribution for *Col14a1* and *Mest* across pseudotime. Both genes are part of cluster pC1 and mark the beginning points of RNA velocity Path1 and Path2. Expression levels for each cell are color-coded with the highest expression level colored black. **e** Feature plots of expression distribution for contractile markers *Acta2* and *Tagln* across pseudotime. Cells with the highest expression level are colored black. Numbers of both *Acta2*^*+*^ and *Tagln*^*+*^ cells peak around minor bifurcations and along Path3. **f**, **g** Expression level heatmaps for selected signaling factors (**f**) and transcription factors (**g**) identified as differentially expressed on **c**. Each heatmap is accompanied by color-coded pseudotime feature plots for two selected genes. Numbers of *Ogn*^*+*^ and *Klf4*^*+*^ cells peak at the beginning points of RNA velocity Path1 and Path2, while numbers of *Pdgfa*^*+*^ and *Ebf1*^*+*^ cells increase along Path3. Cells with the highest expression level are colored black

While t-SNE analysis helped to reveal heterogeneity among wound fibroblasts, we also wondered if they share common differentiation trajectories. Indeed, upon wounding, many fibroblasts differentiate into myofibroblasts and on scRNA-seq contractile markers were detected across multiple fibroblast subclusters. In large wounds, myofibroblasts also serve as the principal progenitors for fat regeneration^5^. Ordering of cells in pseudotime^31^ arranged most of fibroblasts into a major trajectory, with two minor bifurcations (Fig. 3a, top). Fibroblasts from different subclusters distributed broadly across the pseudotime space, with *Pdgfra*^*low*^ sC3/9/11 cells primarily occupying the right half of the major trajectory, with the remaining half consisting of *Pdgfra*^*high*^ cells (Fig. 3a, bottom). Next, we performed RNA velocity analysis, which considers both spliced and unspliced mRNA counts to predict potential directionality and speed of cell state transitions. It represents such transitions as vectors, with long vectors marking rapid differentiation events^32^. RNA velocity distinguished three sets of vectors, defined as paths. Path1 and Path2 vectors appear to represent differentiation of the *Crabp1*^*neg*^ fraction of *Pdgfra*^*high*^ cells and a fraction of *Pdgfra*^*low*^ cells, respectively, toward a shared state surrounding minor bifurcations, that is primarily composed of *Crabp1*^*+*^ *Pdgfra*^*high*^ fibroblasts. Path3 vectors appear to represent differentiation of mainly *Pdgfra*^*low*^ fibroblasts toward a distinct state (Fig. 3b). Cells expressing collagen *Col14a1* and mesoderm-specific transcript *Mest* preferentially distributed at the beginning of all paths (Fig. 3d; Supplementary Data 3), while the contractile genes *Acta2* and *Tagln* increased in density and expression levels toward the ends of these paths (Fig. 3e). We posit that all three RNA velocity paths may represent putative fibroblast differentiation trajectories toward myofibroblast states. We also examined pseudotime dynamics using scEpath^33^, which identifies pseudotime-dependent genes and arranges them into clusters. This analysis revealed five gene clusters (Fig. 3c), including multiple signaling factors and TFs (Fig. 3f, g; Supplementary Figure 10). Overlaying RNA velocity vectors onto these clusters suggests that cluster pC1 contains genes expressed at the beginning points of Path1 and Path2, cluster pC2 primarily contains Path1 differentiation program genes, cluster pC3—mainly Path2 differentiation program genes, whereas clusters pC4 and pC5 are largely composed of Path3 differentiation program genes. Taken together, pseudotime, RNA velocity, and scEpath analyses establish a basis for exploring signaling and transcriptional regulators of wound myofibroblast differentiation programs.

### Wounds contain myofibroblasts with hematopoietic features

**Fig. 4:**
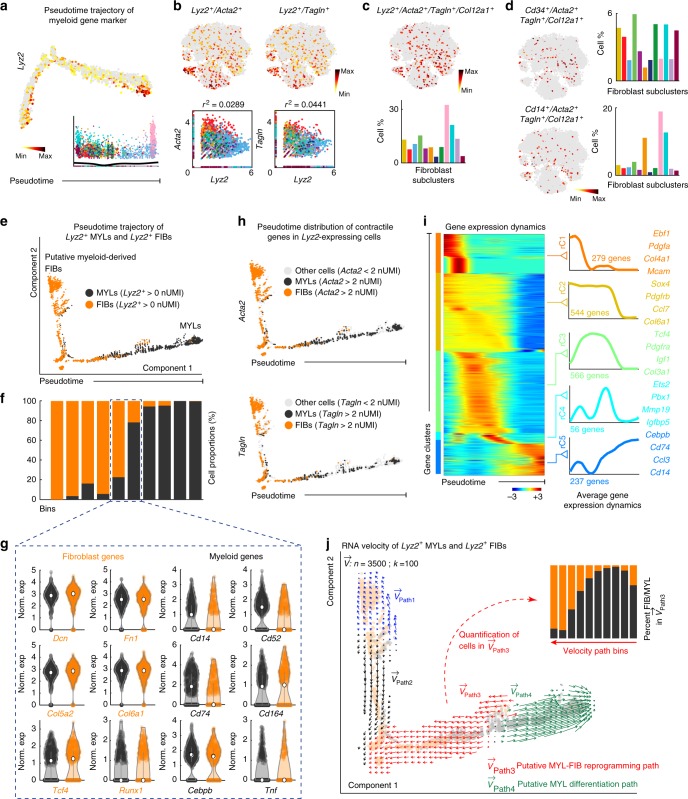
Identification of rare myeloid-derived myofibroblasts in day 12 wounds. **a** Expression levels of myeloid marker *Lyz2* shown as pseudotime feature plot. Cells with the highest expression level are colored black. *Lyz2*^*+*^ cells are present throughout the pseudotime. **b** Overlay of *Lyz2* and contractile markers *Acta2* and *Tagln* expression onto the t-SNE space reveals *Lyz2*^*+*^*/Acta2*^*+*^ and *Lyz2*^*+*^*/Tagln*^*+*^ double-positive wound fibroblasts. **c** Overlay of *Lyz2, Acta2, Tagln* and fibroblast marker *Col12a1* onto the t-SNE space reveals *Lyz2*^*+*^*/Acta2*^*+*^*/Tagln*^*+*^*/Col12a1*^*+*^ quadruple-positive cells among wound fibroblasts. Quantification (bottom) shows that quadruple-positive cells are present in all wound fibroblast subclusters, with subclusters sC9 (pink) and sC10 (teal) being the most enriched. **d** Overlay of *Acta2, Tagln* and *Col12a1* expression with that of the hematopoietic progenitor-associated marker *Cd34* and myeloid marker *Cd14* reveals *Cd34*^*+*^*/Acta2*^*+*^*/Tagln*^*+*^*/Col12a1*^*+*^ and *Cd14*^*+*^*/Acta2*^*+*^*/Tagln*^*+*^*/Col12a1*^*+*^ quadruple-positive cells present among wound fibroblasts. **e** Joint pseudotime ordering on *Lyz2*^*+*^ wound myeloid cells (black) and *Lyz2*^*+*^ fibroblasts (orange). Cells with normalized UMI (nUMI) counts > 0 were selected for this analysis. **f** Stacked bar chart shows relative distribution of fibroblasts and myeloid cells across the pseudotime, which was divided into 10 equal bins. **g** Middle two bins show almost equal mixing of both cell types with hybrid expression patterns, simultaneously enriched for established fibroblast-specific and myeloid-specific genes. **h** Distribution of cells expressing contractile markers *Acta2* and *Tagln* shown as pseudotime feature plots. Cells were considered positive if their nUMI counts were > 2. Positive fibroblasts are colored orange, positive myeloid cell—black and negative cells—light gray. Positive cells are present across the entire pseudotime. **i** scEpath analysis performed on pseudotime from **e** along the fibroblast-to-myeloid trajectory identified five gene clusters (rC1 through rC5) of pseudotime-dependent genes. Heatmap is shown on the left. Average gene expression, gene counts and selected genes in all five clusters are shown on the right. **j** RNA velocity analysis distinguished several sets of velocity vectors across the pseudotime. Path3 vectors (red) encompass the middle bins from **g** and appear to represent putative transition of *Lyz2*^*+*^ myeloid cells into fibroblasts. Path4 vectors (green) appear to mark differentiation of wound-resident myeloid cells. FIB: fibroblast, MYL: myeloid cell

We noted that, on scRNA-seq, many fibroblasts across all 12 subclusters expressed hematopoietic markers. Specifically, many fibroblasts expressed the myeloid-specific marker *Lyz2 (Lysozyme 2*, aka *LysM)* (Fig. 4a). We examined fibroblasts that co-expressed *Lyz2*, collagen *Col12a1* and contractile markers *Acta2* and *Tagln* (Fig. 4b, c). This identified *Lyz2*^*+*^*/Acta2*^*+*^*/Tagln*^*+*^*/Col12a1*^*+*^ quadruple-positive myofibroblasts, representing ~11% of all wound fibroblasts. They were present in all subclusters with sC9 and sC10 being the most enriched, containing ~32% and ~21% of such cells, respectively (Fig. 4c). We also used two additional markers, hematopoietic progenitor-associated *Cd34* and myeloid-specific *Cd14* to identify presence of *Cd34*^*+*^*/Acta2*^*+*^*/Tagln*^*+*^*/Col12a1*^*+*^ and *Cd14*^*+*^*/Acta2*^*+*^*/Tagln*^*+*^*/Col12a1*^*+*^ quadruple-positive myofibroblasts (Fig. 4d).

To examine if *Lyz2*^*+*^ myofibroblasts might derive from wound-resident myeloid cells, we performed pseudotime analysis jointly on all *Lyz2*-expressing wound fibroblasts and myeloid cells (cells from cluster C3 on Fig. 1b). This analysis arranged all cells into a major trajectory, with several minor bifurcations (Fig. 4e). Fibroblasts (orange) and myeloid cells (black) distributed differentially across the pseudotime space; however, a prominent overlap region was observed in the middle of the trajectory (Fig. 4f). Within this region, both cell types displayed hybrid expression patterns, simultaneously enriched for fibroblast-specific and myeloid-specific genes (Fig. 4g; Supplementary Figure 11a). *Acta2* and *Tagln* were enriched in *Lyz2*^*+*^ fibroblasts, but were also expressed in *Lyz2*^*+*^ myeloid cells, including in the overlap region (Fig. 4h). scEpath identified five pseudotime-dependent gene clusters (Fig. 4i; Supplementary Figure 11b–e; Supplementary Data 4), while RNA velocity distinguished four vector paths (Fig. 4j). Importantly, Path3 vectors, which encompass the overlap region, appear to represent putative conversion of *Lyz2*^*+*^ myeloid cells into fibroblasts (red on Fig. 4j), while Path4 vectors appear to mark differentiation of wound-resident myeloid cells (green on Fig. 4j). Overlaying RNA velocity vectors onto scEpath data indicates that genes in cluster rC5 mark myeloid differentiation program along Path4, while genes in clusters rC4 and rC3 mark early and late stages of putative myeloid-to-fibroblast conversion pathway, respectively.

**Fig. 5:**
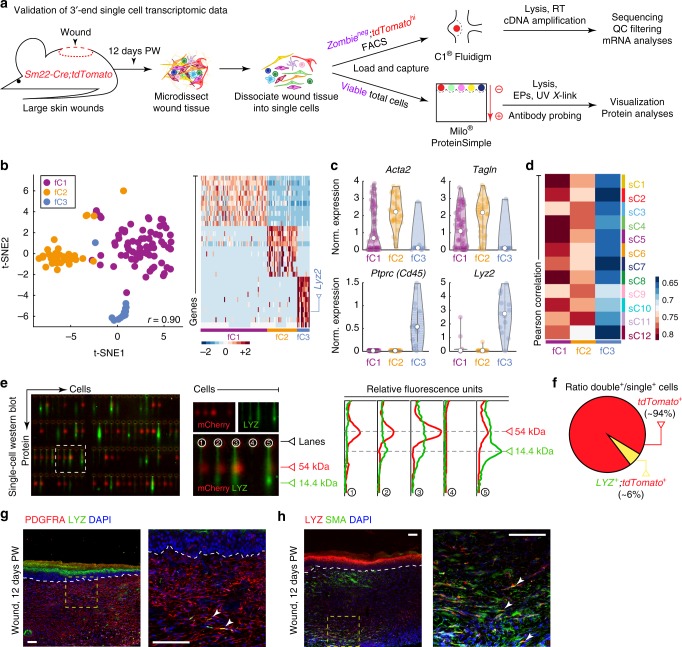
Validation of myeloid-derived myofibroblasts in day 12 wounds. **a** Schematic of cell isolation from day 12 wounds in *Sm22-Cre;tdTomato* mice, sorting for tdTomato^+^ cells, capture by microfluidic device, sequencing and downstream analysis, and wound cell processing for single-cell western blot. **b** t-SNE plot revealed heterogeneity among tdTomato^+^ wound cells. 116 sequenced cells were analyzed and three clusters were identified. Heatmap of top differentially expressed genes is shown on the right and *Lyz2* is marked. **c** Violin plots of contractile markers *Acta2* and *Tagln*, pan-hematopoietic marker *Ptprc* (aka *Cd45*) and myeloid marker *Lyz2*. **d** Pearson correlation analysis between day 12 tdTomato^+^ cell clusters and day 12 wound fibroblasts subclusters from Fig. 2a. **e** Single-cell western blot on unsorted cells from day 12 *Sm22-Cre;tdTomato* wounds revealed LYZ-expressing myofibroblasts. Chips were co-stained for LYZ (green) and tdTomato (mCherry, red). Graph on the right shows LYZ^+^/tdTomato^+^ double-positive cells in lanes #2, #3, and #5. Cell in lane #1 is single-positive for tdTomato and cell in lane #4 is double-negative. **f** Quantification shows that ~6% of tdTomato^+^ wound cells co-express LYZ (77 out of 1293 cells). See also Supplementary Figure 14. **g** Co-staining of day 12 wounds for LYZ (green) and PDGFRA (red) identified occasional double-positive fibroblasts. **h** Co-staining of day 12 wounds for LYZ (red) and SMA (green) identified occasional double-positive myofibroblasts. Immunostaining images shown on **g** and **h** are representative of the marker staining patterns observed in three or more independently stained samples. PW: post-wounding, FACS: fluorescence-activated cell sorting, RT: reverse transcription, EP: electrophoresis, QC: quality control. Size bars: **g**, **h**—100 µm

Next, we performed scRNA-seq on genetically marked contractile cells, isolated from day 12 wounds of *Sm22-Cre;tdTomato* mice (Fig. 5a). A microfluidic-based capture platform was used (Supplementary Figure 12a). Although it captured substantially fewer cells, 116 in total, compared to the droplet-based capture approach, it yielded a higher depth of sequencing, 5830 vs. 1101 median genes/cell (Supplementary Figure 12b vs. 2b), and eliminated the possibility of cell doublets. On t-SNE analysis, cells segregated into three clusters—fC1, fC2, and fC3 (Fig. 5b), each with its distinct gene markers (Supplementary Figure 13; Supplementary Data 5). Pearson correlation showed that fC1 cells were the most similar to *Pdgfra*^*high*^ fibroblasts from subclusters sC1/4/5/8/10. Cells from fC2 were the most similar to *Pdgfra*^*low*^ fibroblasts from subclusters sC3/11 and expressed high levels of *Pdgfrb*. Cluster fC3 had the highest correlation to sC9 subcluster (Fig. 5d), enriched for *Lyz2*^*+*^*/Acta2*^*+*^*/Tagln*^*+*^*/Col12a1*^*+*^ quadruple-positive myofibroblasts. Consistently, this cluster was also the most enriched for hematopoietic markers, including pan-hematopoietic *Cd45* (aka *Ptprc*), and myeloid-specific *Lyz2* and *Ccl6* (Fig. 5c; Supplementary Figure 13). To confirm *Lyz2*^*+*^ myofibroblasts at the protein level, we performed single-cell western blot on unsorted cells from day 12 *Sm22-Cre;tdTomato* wounds and stained for LYZ and tdTomato (Fig. 5e; Supplementary Figure 14). Indeed, ~6% of tdTomato-expressing wound cells were LYZ^+^/tdTomato^+^ double-positive (77 out of 1293 cells) (Fig. 5f). We also co-stained day 12 wounds for LYZ and fibroblast markers PDGFRA (Fig. 5g) and SMA (Fig. 5h), and in both cases detected double-positive cells.

To determine if myeloid marker-expressing fibroblasts persist in healed wounds, we performed scRNA-seq on tdTomato^hi^ cells isolated from *Sm22-Cre;tdTomato* wounds at two additional time points, 15 days and 21 days PW (Supplementary Figure 15a). Combined analysis on all cells from 12 days, 15 days, and 21 days PW showed that they form four clusters, tC1 through tC4 (Supplementary Figure 15b; Supplementary Data 6). Cells from all three time points contributed to all four clusters (Supplementary Figure 16a), although relative cluster contribution was time point-dependent (Supplementary Figures 15c and 16b). Cluster tC4 was closely related to cluster fC3 from 12 days PW analysis (Supplementary Figure 15d) and also contained *Cd45* and *Lyz2* expressing cells (Supplementary Figure 16c). Taken together, we identified a population of myofibroblasts with myeloid features that persist in wounds over the course of repair and regeneration and potentially form via myeloid cell reprograming.

### Hematopoietic lineage cells contribute to wound dermis

**Fig. 6:**
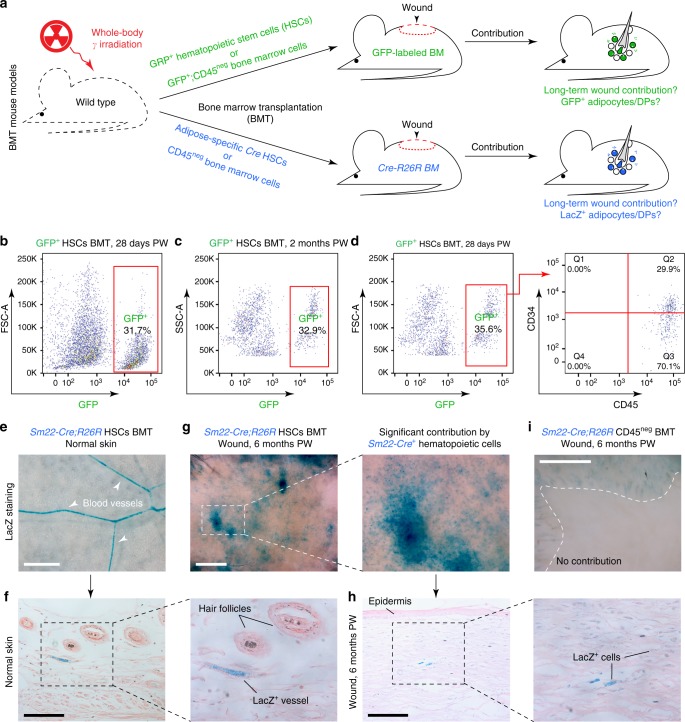
Hematopoietic lineage contributes toward regenerating wounds in BMT mice. **a** Schematic representation of the key BMT experiments used to assess the contribution of hematopoietic lineage cells to de novo adipocytes. **b–d** Based on flow cytometry analysis, the hematopoietic lineage contributes ~30% of the cells in dermal wound tissue 28 days and 2 months PW in GFP^+^ HSCs BMT mice. **e**–**h** LacZ expression patterns in the wounds of *Sm22-Cre;R26R* HSCs BMT mice confirmed that a portion of contractile wound cells (**g**, **h**) and perivascular contractile cells (arrowheads on **e**) originate from the hematopoietic lineage. **i** Wounding experiments in *Sm22-Cre;R26R* CD45^neg^ BMT mice showed that non-hematopoietic BM cells do not contribute toward contractile wound and peri-wound cells. Images shown in **e–i** are representative of the lacZ expression patterns observed in three or more independent samples. HSC: hematopoietic stem cell, BM: bone marrow, BMT: bone marrow transplantation, DP: dermal papilla, PW: post-wounding day. Size bars: **e**, **g**, **i**—1 mm; **f**, **h**—125 µm

**Fig. 7:**
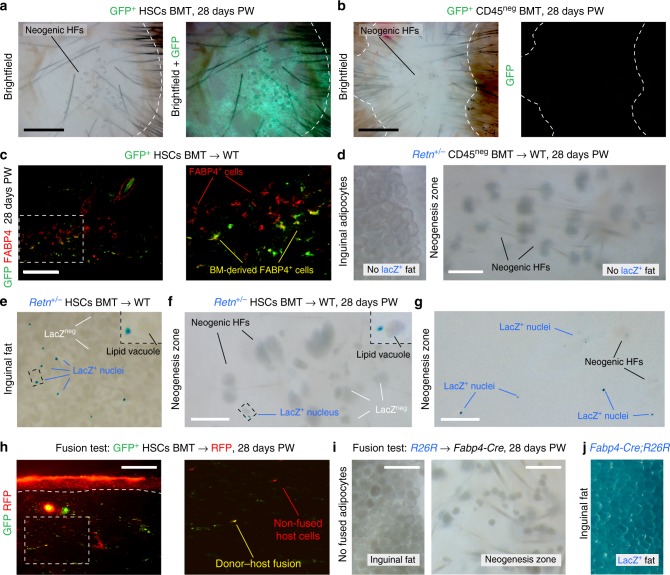
Hematopoietic lineage cells contribute to rare de novo adipocytes. **a**, **b** GFP expressing cells in GFP^+^ HSCs BMT mice (**a**), but not in GFP^+^ CD45^neg^ BMT mice (**b**), showed significant contribution to the areas of neogenesis 28 days PW. See also Supplementary Figure 19. **c** In the wounds of GFP^+^ HSCs BMT mice, GFP^+^ cells localize around neogenic hair follicles and a portion of them co-expresses FABP4 at 28 days PW. Image is representative of the marker staining pattern observed in three or more independently stained samples. **d** CD45^neg^ BM cells do not contribute toward adipocytes in the inguinal fat or regenerated 28 days PW wounds of BMT mice reconstituted with *Retn-lacZ* CD45^neg^ cells. **e**–**g** In contrast, hematopoietic lineage cells consistently contributed toward rare adipocytes (cells with nuclear lacZ expression) in the inguinal fat (**e**) and regenerated 28 days PW wounds (**f**, **g**) of BMT mice reconstituted with *Retn-lacZ* HSCs. Insets on **e** and **f** show positive adipocytes with lipid vacuole and peripherally-positioned lacZ^+^ nucleus. **h** Only rare cell fusion events were identified on the basis of GFP/RFP double fluorescence in 28 days PW wounds of GFP^+^ HSCs into RFP^+^ BMT mice. Fused and non-fused host cells are marked on the enlarged inset. Image is representative of the GFP and RFP expression patterns observed in three or more independently stained samples. **i**, **j** Lack of cell fusion-dependent de novo adipocytes in wounds of BMT models was confirmed by the absence of lacZ^+^ cells in *R26R* HSCs into *Fabp4-Cre* BMT mice (**i**). This was contrasted by robust lacZ expression in the inguinal fat of *Fabp4-Cre;R26R* mice (**j**). PW: post-wounding, BM: bone marrow, BMT: bone marrow transplantation, HSC: hematopoietic stem cell, HF: hair follicle. Size bars: **a**, **b**—1 mm; **c**, **h**—50 µm; **d**, **f**, **g**, **i**—200 µm

Following our scRNA-seq findings, we noted that previous work indicated that myeloid cells can convert into myofibroblasts^34^, but the extent of this conversion is organ and injury context-specific^35–41^. Considering that in large wounds, adipocytes regenerate predominantly from myofibroblasts^5^, we asked to what extent myeloid cells contribute to their population. We performed wounding experiments in bone marrow transplantation (BMT) mouse models. In some BMT models, we reconstituted lethally irradiated mice with GFP-expressing cells, while in others with cells expressing lacZ under the control of lineage-specific promoters (Fig. 6a). To distinguish between hematopoietic and non-hematopoietic cells, we performed BMT using the sorted CD45^+^ bone marrow (BM) fraction. In CD45^+^ BMT models, we achieved high levels of peripheral blood chimerism, compatible to BMT mice reconstituted with whole BM (Supplementary Figure 17). We also generated BMT mice using multipotent hematopoietic stem cells (HSCs) purified based on the SLAM marker signature: Lineage^neg^, SCA1^+^, c-KIT^+^, CD150^+^, CD48^neg^ (Supplementary Figure 17e). We transplanted between 2300 and 4400 HSCs per recipient mouse and in all cases achieved successful reconstitution of the hematopoietic lineage, confirmed by high levels of peripheral blood chimerism (Supplementary Figure 17f, g) and bone marrow fluorescence (Supplementary Figure 18a). Following wounding, healed tissue in GFP^+^ HSCs BMT mice showed consistently high contribution from hematopoietic lineage on 28 days PW, with many GFP^+^ cells surrounding neogenic hair follicles (*n* = 18 animals) (Fig. 7a; Supplementary Figure 19). Flow cytometry on wound tissue confirmed that long-term contribution from the hematopoietic lineage constituted ~30% at both 28 days (*n* = 3 biologically independent samples) and 2 months PW (*n* = 3 biologically independent samples) (Fig. 6b–d). In contrast, BMT mice that received GFP^+^ CD45^neg^ non-hematopoietic BM fraction, which includes mesenchymal stromal cells, had no GFP^+^ contribution to the wound (Fig. 7b) despite showing BM repopulation (*n* = 11 animals; these BMT mice also received wild type whole BM support cells) (Supplementary Figure 18b).

Next, we wounded BMT mice reconstituted with *Sm22-Cre;R26R* HSCs. In this model, we expected lacZ expression to mark contractile cells that derived from reconstituted hematopoietic lineage cells (but not from transplanted HSCs directly), that activated *Sm22* promoter-controlled Cre recombinase. Indeed, consistent with the possibility of long-lasting hematopoietic contribution to wound myofibroblasts we observed lacZ-positive cells in wounds from *Sm22-Cre;R26R* HSCs BMT mice (*n* = 9 animals) 6 months after wounding (Fig. 6e–h). This was contrasted by the absence of lacZ-positive cells in the wounds of BMT mice reconstituted with *Sm22-Cre;R26R* CD45^neg^ BM cells (*n* = 4 animals) (Fig. 6i). We also found lacZ-expressing cells along many blood vessels (Fig. 6e, f), suggesting that upon BMT hematopoietic lineage cells may also participate in reconstituting perivascular mural cells.

### Hematopoietic lineage cells convert to de novo adipocytes

Because fibroblast and fat lineages are closely related developmentally, we hypothesized that some hematopoietic lineage cells that initially convert into myofibroblasts might then become de novo adipocytes. Although contested^42,43^, several studies have reported that, in principle, myeloid cells can convert into adipocytes upon integration into pre-existing fat tissue^44–46^. Contribution of myeloid cells towards fat is variable and is dependent on gender and anatomical site, with visceral fat in female mice experiencing up to 10% contribution^44^.

We employed both BMT and non-BMT mouse models to evaluate this possibility. Indeed, wounds in GFP^+^ HSCs BMT mice contain many GFP^+^ cells that co-stained for the adipocyte marker FABP4 in areas surrounding neogenic hair follicles (Fig. 7c). By taking advantage of the fact that in mice the expression of *Retn*, which encodes for Resistin, is exclusive to mature fat cells, we generated *Retn-lacZ* HSCs BMT and *Retn-lacZ* CD45^neg^ BMT models using the approach described above (Fig. 6a). In these mice, nuclear lacZ expression can be used to genetically identify functionally mature de novo adipocytes that may have derived from transplanted BM progenitors rather than from the host sources. We consistently observed formation of occasional lacZ-positive de novo adipocytes in the hair-bearing wounds of *Retn-lacZ* HSCs BMT mice (*n* = 72 animals) (Fig. 7f, g), but not in *Retn-lacZ* CD45^neg^ BMT mice (*n* = 50 animals) (Fig. 7d). Importantly, in agreement with a previous report^44^, we also observed integration of hematopoietic-derived lacZ-expressing adipocytes into normal fat of *Retn-lacZ* HSCs BMT mice (Fig. 7e).

Next, we examined if the hematopoietic lineage in BMT mice contributes to de novo adipocytes via direct conversion rather than cell fusion. For that purpose, we generated BMT models in which we transplanted GFP^+^ HSCs into RFP^+^ hosts and *R26R*^*+/−*^ HSCs into *Fabp4-Cre* hosts. In the first model, all fused cells can be identified on the basis of GFP/RFP double fluorescence. In the second model only fusion-derived de novo adipocytes contain *Fabp4-Cre* construct necessary for *R26R* reporter activation. We observed only very occasional GFP/RFP double positive cells in the hair-bearing wounds of GFP^+^ HSCs into RFP^+^ BMT mice (*n* = 8 animals) (Fig. 7h). LacZ-positive adipocytes were not observed in regenerated wounds and in normal fat of *R26R*^*+/−*^ HSCs into *Fabp4-Cre* BMT mice (*n* = 13 animals) (Fig. 7i), in contrast to robust lacZ expression in fat of non-BMT *Fabp4-Cre;R26R* mice (Fig. 7j). These experiments confirm that hematopoietic-to-adipose lineage conversion, rather than cell fusion, takes place during de novo fat regeneration in the wound.

### Lineage study confirms myeloid origin of de novo adipocytes

**Fig. 8:**
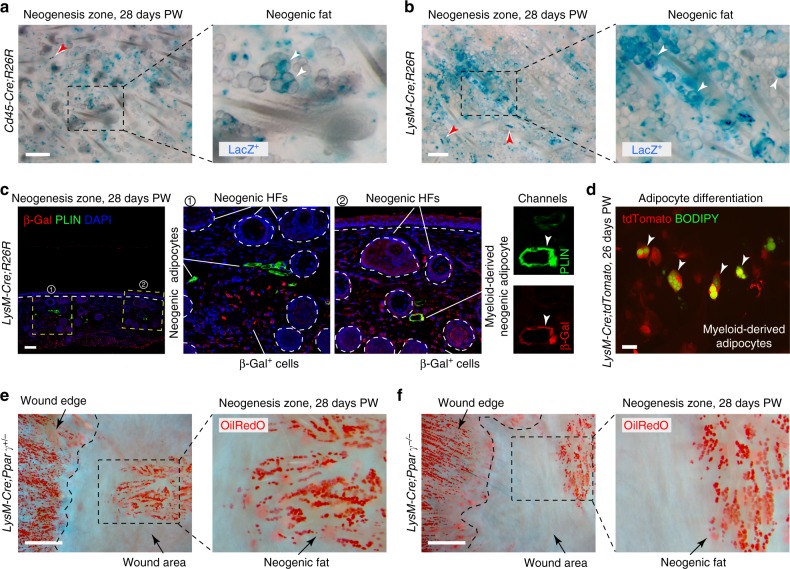
Myeloid lineage cells contribute to rare de novo adipocytes. **a**, **b** LacZ^+^ clusters of de novo adipocytes (white arrowheads) were consistently identified in the wounds of pan-hematopoietic specific *Cd45-Cre;R26R* (**a**) and myeloid-specific *LysM-Cre;R26R* mice (**b**). Red arrowheads mark lacZ^+^ DPs of neogenic hair follicles. See also Supplementary Figure 20. **c** Co-staining of day 28 *LysM-Cre;R26R* wounds for β-Gal (red) and PLIN (green) identified both single-positive (inset 1) and double-positive mature adipocytes (inset 2) in the region of neogenesis. Image is representative of the marker staining patterns observed in three or more independently stained samples. **d** Lipid-laden adipocytes (BODIPY^+^, green) can be differentiated in vitro under adipogenic conditions from tdTomato^+^ cells (red), isolated from day 26 hair-bearing wounds from *LysM-Cre;tdTomato* mice. **e**, **f** In agreement with the limited lineage contribution from myeloid cells toward de novo adipocytes *LysM-Cre;Ppar*γ^−/−^ mutants regenerated nearly normal-looking de novo fat (compare mutant wound on **f** with heterozygous control on **e**). Adipocytes were stained red with OilRedO. PW: post-wounding, HF: hair follicle. Size bars: **a**, **b**—200 µm; **c**—100 µm; **d**—25 µm; **e**, **f**—1 mm

We then studied wound healing in *Cd45-Cre;R26R* mice to verify that hematopoietic cells contribute to de novo fat physiologically and not only in the context of BMT models. In these mice, where Cre activity is restricted to the hematopoietic lineage^47^, we observed consistent, albeit occasional formation of lacZ-positive de novo adipocytes (*n* = 9 animals) (Fig. 8a; Supplementary Figure 20a). Similarly, lacZ-positive adipocytes formed in the wounds of *LysM-Cre;R26R* mice (*n* = 12 animals) (Fig. 8b, c; Supplementary Figure 20b), and a portion of tdTomato^+^ cells, derived from 26 days PW hair-bearing wounds of *LysM-Cre;tdTomato* mice, differentiated into lipid-laden adipocytes when cultured under adipogenic conditions (*n* = 3 replicate cultures) (Fig. 8d). This suggests that hematopoietic lineage contribution to fat neogenesis is mediated via myeloid progenitors. Consistent with occasional distribution patterns of lacZ-positive adipocytes in wounds of *LysM-Cre;R26R* mice, *LysM-Cre;Pparγ*^−/−^ mutants did not have a clear de novo fat defect (*n* = 20 animals) (Fig. 8e vs. 8f), characteristic of *Sm22-Cre;Pparγ*^−*/*−^ mice described in Plikus, Guerrero-Juarez^5^. Curiously, in both mouse models, we also observed occasional formation of neogenic hair follicles with lacZ-positive DPs and dermal sheath (Fig. 8a, b, red arrowheads; Supplementary Figure 20), suggesting that the lineage plasticity repertoire of myeloid cells during wound regeneration might extend beyond adipogenesis. Taken together, our lineage tracing studies help to establish the role of myeloid cells as one of the sources of adipogenic progenitors during wound regeneration.

## Discussion

Traditionally, adult mammals are considered to have limited regenerative abilities and scarring is thought to be the default repair response. The notable exceptions to this rule are digit tip regeneration after amputation^48,49^ and neogenesis of hair follicles^4,6,7,14^ and fat^5^ in the center of large excisional wounds. Intriguingly, lineage studies reveal important differences in the regenerative strategies between these two systems. Epithelial and mesenchymal structures in the digit tip regenerate from several types of fate-restricted progenitors and no multipotent progenitors or lineage reprogramming events are observed^48,49^. In contrast, large skin wounds demonstrate broadened lineage plasticity. Although progeny of preexisting hair-fated bulge stem cells migrate into wound epidermis, they do not partake in hair follicle neogenesis^4^. Instead, new hair follicles regenerate from non-bulge epithelial stem cells^15,20^, among other sources. Fat neogenesis is driven by lineage reprogramming of non-adipogenic wound myofibroblasts^5^. DP neogenesis also likely relies on myofibroblast reprogramming strategy^14^.

Are all wound myofibroblasts identical or heterogeneous in terms of their origin, properties, and morphogenetic competence? While studying cell origin requires genetic fate mapping, and assessment of morphogenetic competence requires functional studies, scRNA-seq allows large-scale profiling of cell properties in complex tissues. Recently, this approach has been applied to studying cellular heterogeneity in the skin, including epithelial cells of mouse hair follicles^50,51^, and early wounds^52^, and keratinocytes^53^ and fibroblasts^54,55^ of human skin. Tabib, Morse^55^ identified two major populations of human skin fibroblasts, characterized by co-expression of *SFRP2*^*+*^*/DPP4*^*+*^ and *FMO1*^*+*^*/LSP1*^*+*^ markers. These further subdivide into several subpopulations. Philippeos, Telerman^54^ identified five fibroblast populations: upper and lower dermal fibroblasts, pericytes, and two uncharacterized populations. scRNA-seq has also been used to study heterogeneity of disease-associated fibroblasts in the synovial tissue upon rheumatoid arthritis^56,57^ and in the lung upon bleomycin-induced fibrosis^58^.

Here, we studied fibroblast heterogeneity in the mouse model for wound-induced regeneration at 12 days PW when wound re-epithelialization is completed and preceding hair follicle neogenesis. At later time points 15 days and 21 days PW, we anticipated a decrease in SMA expression and change in fibroblast types. We show that wound fibroblasts can be broadly classified into two major populations on the basis of their transcription factor signatures and PDGF receptor expression patterns. *Pdgfra*^high^ fibroblasts, which dominate in the wound, further subdivided into upper and lower dermal fractions, based on their localization and gene expression. *Pdgfra*^low^ cells constituted nearly a quarter of all wound fibroblasts and localized throughout wound dermis. Lineage tracing will be required to determine which fibroblast population preferentially contributes toward newly regenerating hair follicle mesenchyme and adipocytes. Prominent additional heterogeneity exists within both populations, and pseudotime and RNA velocity analyses predict three distinct fibroblast-to-myofibroblast differentiation trajectories. Future work should aim to understand the functional significance of this heterogeneity in the context of regeneration and to lineage trace wound fibroblast origin to distinct skin fibroblasts in the unwounded skin.

BM-derived progenitors, including myeloid cells, endothelial progenitors, and circulating mesenchymal stem cells can contribute new stromal cells toward injured tissues in various organs. Scar tissue in heart following myocardial infarction^59^, cornea following keratectomy^60^, and lung in pulmonary fibrosis^61^ contains BM-derived collagen-producing myofibroblasts. In skin, studies document BM giving rise to fibroblasts at the injury sites^35–41^, while some studies report this contribution to be minimal^62,63^. Such a discrepancy is likely attributed to several factors, including the type and extent of injury and experimental timing. The contribution from BM progenitors toward repairing tissues was shown to increase with the extent of injury^37,64,65^, yet most of the previous studies were performed on small wounds. Also, many previous studies failed to evaluate long-term BM contribution, yet BM-derived fibroblasts in the scar were shown to peak after 22 days^35^.

Our data from large excisional wounds shows that the contribution from myeloid cells to wound fibroblasts is small yet significant, between 6% and 11.3%, depending on the assessment method. scRNA-seq analysis revealed an intermediate state of wound cells, displaying dual gene expression signature, enriched for contractile and mesenchymal extracellular matrix genes, as well as myeloid cell surface antigens and inflammatory cytokines. RNA velocity analysis showed that wound cells progress through this intermediate state in a myeloid-to-fibroblast direction. We also showed that at least a portion of these cells can convert into de novo adipocytes around neogenic hair follicles. Previously, adipogenic conversion of myeloid cells has been shown both in vitro^66,67^ and in vivo in major adipose depots, such as in inguinal fat^44,45^; although some studies contested these observations^42,43^. Most recently, ~10% of adipocytes were shown to form from hematopoietic lineage in human subjects undergoing BMT treatment^46^, while in another human BMT study, up to 35% of adipocytes were traced to transplanted BM sources^45^.

Overall, our findings illustrate the dynamic nature of fibroblast identities during wound healing, and the powerful wound-induced plasticity of myeloid lineage cells. scRNA-seq analysis infers several pathways fibroblasts follow during wound healing, including contractile and regenerative. The lineage relationship between myeloid cells and adipogenesis suggests further characterization of the factors influencing plasticity and fate switching could uncover potential therapeutic approaches to treating wounds and scars.

## Methods

### Mouse models

The following mouse models were used in this study: *C57BL/6J* (JAX stock 000664), *Retn-lacZ*, *Sm22-Cre* (JAX stock 004746), *Cd45-Cre*, *LysM-Cre* (JAX stock 004781), *Fabp4-Cre* (JAX stock 005069), *Pparγ*^*flox*^ (JAX stock 004584), *R26R* (JAX stock 003474), *tdTomato* (JAX stock 007909), *GFP* (JAX stock 004353), *RFP* (JAX stock 006051), *and Rag1*^*−/−*^ (JAX stock 002216).

### Wounding experiments

All animal experiments were carried out in accordance with the guidelines of the Institutional Animal Care and Use Committee of the University of Pennsylvania and University of California, Irvine. Animals were anesthetized with isoflurane, hairs were clipped, skin site was disinfected and a single full thickness excisional wound was created on their dorsum using scissors (squared *s* *=* 1.5 cm)^4–6^. Both male and female mice between 6 and 8 weeks of age were used. Following wounding, all animals were housed individually. Wounds were let to heal by secondary intention. No wound dressing was applied. Animals were used as biological replicates.

### Bone marrow transplantation

Bone marrow cells were flushed from the long bones with Dulbecco’s Modified Eagle’s Medium supplemented with 5% heat-inactivated calf serum (Gibco). To obtain a single cell suspension, cells were filtered through a 45 µm nylon screen. Cells were blocked with anti-rat and anti-mouse IgG (Sigma) for 15 min. For isolation of HSCs, cells were then stained with CD45 (APC-Cy7, BD Pharmingen), CD150 (APC, Biolegend), CD48 (Pacific Blue, Biolegend), SCA1 (PE-Cy5.5, Invitrogen), and c-KIT (PE-Cy7, eBioscience), as well as PE-conjugated lineage markers including CD19, B220, CD3, CD4, CD8, GR-1, MAC-1, NK1.1, and TER119 (eBioscience). Cells were resuspended in DAPI to discriminate live from dead cells. HSCs were sorted by isolating Lineage^neg^, SCA1^+^, c-KIT^+^, CD150^+^, CD48^neg^ cells using a BD FACSAria II flow cytometer (BD Biosciences). Approximately 150,000–2,000,000 CD45^+^ cells or 2300–4400 HSCs, or ~150,000–2,000,000 CD45^neg^ cells were resuspended in phosphate buffered saline with 100,000–200,000 whole bone marrow cells for reconstitution and injected into lethally irradiated isogenic wild type host mice. For *Sm22-Cre;R26R* BMT experiments, cells were transplanted into immunodeficient *Rag1*^*−/−*^ mice. Donor chimerism was assessed by measuring GFP or CD45.1 vs. CD45.2 levels by FACS in the peripheral blood 8 weeks after transplantation.

### Whole mount lacZ staining

To detect lacZ activity, freshly isolated wound tissue samples were incubated with X-gal reagent in lacZ staining buffer^4,5^. Samples were post-fixed in 4% PFA.

### Whole mount OilRedO staining

PFA-fixed wound tissue samples were pre-incubated in propylene glycol and then stained with OilRedO buffer for 20 min. Samples were then washed in propylene glycol and stored in 0.05% aqueous solution of sodium azide.

### Histology and immunohistochemistry

Tissues were fixed in 4% PFA, dehydrated, paraffin embedded, and sectioned at 7–12 µm thickness. Frozen tissues were sectioned at 12 µm thickness. Immunostaining was performed both on frozen and paraffin sections. Heat-based antigen retrieval was performed when necessary. Tissue sections were blocked in either 3% BSA, 3% Donkey serum, or 3% mouse IgG from Mouse on Mouse (MOM) kit (Vector Labs). The primary antibodies used were goat anti-FABP4 (1:200; R&D Systems), mouse anti-SMA (1:500; R&D Systems), rabbit anti-RGS5 (1:250; Proteintech), rabbit anti-LYZ (1:250; Abcam), rabbit anti-CRABP1 (1:800; Cell Signaling), goat anti-CYP26B1 (1:50; Novus Biologicals); rabbit anti-G0S2 (1:100; Proteintech), mouse anti-MEST (1:250; Proteintech), goat anti-PDGFRA (1:350; R&D Systems), chicken anti-β-Gal (1:500; Abcam), and rabbit anti-PLIN (1:800; Cell Signaling). Tissue sections were visualized with Nikon T inverted (Nikon) and Olympus FluoView confocal (Olympus) microscopes.

### Cell culture and adipocyte differentiation

Primary cells were isolated from 26 days PW pooled wound tissues of *LysM-Cre;tdTomato* mice. Tissues were micro-dissected, minced, and incubated in a Collagenase IV solution for 60 min with constant rotation. Post-incubation, cells were filtered through a 40 µm nylon screen, centrifuged, washed, resuspended in DMEM supplemented with 10% FBS, antibiotics, and antifungals and cultured at 37 °C in a water-jacketed incubator with 5% CO_2_ output. Once cells reached confluency, they were switched to adipocyte differentiation media (Cell Solutions) for 72 h, upon which they were switched to adipocyte maintenance media (Cell Solutions) for additional 7 days. Cells were incubated with BODIPY (Thermo) as per manufacturer’s directions and visualized using a Nikon T inverted (Nikon) microscope.

### 3′-end single cell RNA-sequencing

Pooled wound tissues (*n* = 12 animals) were collected from *Sm22-Cre;tdTomato* mice on 12 days PW. Tissues were micro-dissected, minced, and incubated in a Dispase II/Collagenase IV/Liberase solution for 60 min with constant rotation. Post-incubation, cell aggregates were mechanically dissociated using GentleMACS (MACS). Single cell suspensions were treated with 1X RBC lysis buffer, washed, and re-suspended in 0.04% UltraPure BSA (Biolegend). Dead cells were removed using the MS columns of the Dead Cell Removal Kit (MACS) as per manufacturer’s directions. Live cells were resuspended in 0.04% UltraPure BSA and counted using the automated cell counter Countess (Thermo). GEM generation, barcoding, post GEM-RT cleanup, cDNA amplification, and cDNA library construction were performed using Single Cell 3′ v2 chemistry (10X Genomics). Library quality control metrics were as follows: 550.19 pg/µl with an average size ~454 bps. Libraries were sequenced on an Illumina HiSeq4000 platform (Illumina) (one lane, 100 PE). Cell counting, suspension, GEM generation, barcoding, post GEM-RT cleanup, cDNA amplification, library preparation, quality control, and sequencing were performed at the Genomics High Throughput Sequencing facility at the University of California, Irvine.

### Data processing for 3′-end transcripts

Transcripts were mapped to the mm10 reference genome (GRCm38.91) using Cell Ranger Version 2.1.0. The web summary of Cell Ranger statistics on cells, sequencing and mapping metrics pre-quality control assessment for downstream bioinformatics analyses were as follows—cell metrics: ~22,322 estimated number of cells, ~84.2% fraction reads in cells, ~13,819 mean reads per cell, ~1101 median genes per cell, ~19,070 total genes detected, ~2448 median UMI counts per cell; sequencing metrics: ~308,471,010 total number of reads, ~98.5% valid barcodes; mapping metrics: ~90.4% reads mapped to genome, ~85.5% reads mapped confidently to genome, ~65.9% reads mapped confidently to transcriptome.

### Quality control metrics for 3′-end transcripts

For downstream analyses after initial Cell Ranger metric assessment, low-quality cells were removed to eliminate cell-specific biases. Quality control metrics included keeping cells displaying < 8000 UMI/cell and < 2500 genes/cell, and no more than 8% mitochondrial gene expression. Post-quality control, 21,819 cells remained for downstream bioinformatic analyses.

### Downstream analyses of 3′-end transcripts

Clustering of cells was performed using the Seurat R package^25^. In brief, digital gene expression matrices were column-normalized and log-transformed. To identify cell clusters, principle component analysis (PCA) was first performed on the list of highly variable genes. A set of highly variable genes was then identified by binning the average expression of all genes into evenly sized groups and computing the median dispersion (variance divided by the mean) in each bin. Genes were selected for inclusion in PCA with an average expression > 0.01 and dispersion > 1.0. To identify significant PCs, we used the Jackstraw method (JackStraw function in Seurat). The top 40 PCs were used for clustering with the Louvain modularity-based community detection algorithm to generate cell clusters (FindClusters function, 13 clusters with resolution = 0.45). Marker genes were determined with *p-*value < 0.01 and log(fold-change) > 0.25 by performing differential gene expression analysis between the clusters using the likelihood-ratio test. To present high dimensional data in two-dimensional space, we performed t-SNE analysis using the results of PCA with significant PCs as input. For the subclustering analysis on all wound fibroblasts, we followed the same procedures as above. Briefly, the top 35 PCs were used for clustering and 12 subclusters were obtained with resolution = 0.6.

### Cell-cycle discrimination analyses

We used cell cycle-related genes, including a previously defined core set of 43 G1/S and 54 G2/M genes^29^. For each cell, a cell cycle phase (G1, S, G2/M) was assigned based on its expression of G1/S or G2/M phase genes using the scoring strategy described in CellCycleScoring function in Seurat. Cells in each cell cycle state were also quantified using the which.cells function in Seurat.

### Pseudotime analyses

We subclustered wound fibroblasts based on known fibroblast-specific markers. We used Monocle 2 to infer the pseudotime trajectories of these cells^31^, followed by scEpath^33^ to identify pseudotime-dependent gene expression changes. To model gene expression changes in pseudotime, scEpath first divides the pseudotime into 10 equally spaced bins. Then the expression of each gene in each bin is estimated by the trimean of the expressions of this gene across all the cells located in this bin. Furthermore, scEpath smoothens the average expression of each gene using cubic regression splines. To determine the pseudotime-dependent genes that are significantly changed, we compared the standard deviation of the observed smoothed expressions with a set of similarly permuted expressions by randomly permuting the cell order (1000 permutations in our analyses). We considered all genes with a standard deviation > 0.5 and a Bonferroni-corrected *p-*value below a significance level *α* = 0.01 to be pseudotime dependent. To analyze pseudotime-dependent TFs, we used genes that are annotated in the Animal Transcription Factor Database (AnimalTFDB 2.0)^68^. For the pseudotime analysis of *Lyz2*^*+*^ wound myeloid cells and *Lyz2*^*+*^ fibroblasts, we followed the same procedures.

### RNA velocity analyses

RNA velocity was calculated on the basis of spliced and unspliced transcript reads as previously reported^32^. Based on velocyto pipeline, annotation of spliced and unspliced reads was performed using the Python script velocyto.py on the Cell Ranger output folder. Only cells that were part of the pseudotime were considered in analyses. PCA analysis was performed with Pagoda2^69^ with spliced expression matrix as input, and cell-to-cell distance matrix was calculated using Euclidean distance based on the top 40 principal components. RNA velocity was estimated using gene-relative model with *k*-nearest neighbor cell pooling (*k* = 100). Velocity fields were projected onto the pseudotime space produced by Monocle 2. Parameter *n* sight, which defines the size of the neighborhood used for projecting the velocity, was 3500. For other parameters, we used the standard R implementation of velocyto with default settings.

### Full length single cell RNA-sequencing

Pooled wound tissues (*n* = 2–3 animals) were collected from *Sm22-Cre;tdTomato* mice on 12, 15, and 21 days PW. Wound tissue was micro-dissected and disaggregated into single cells with Dispase II (Sigma) and Collagenase IV (Sigma) at 37 °C with constant rotation. Single cell fraction was stained with Zombie Violet (1:1000; BioLegend) and FACS-sorted as Zombie^neg^;tdTomato^hi^ with a BD FACSAria II flow cytometer (BD Biosciences). Pre-sorted, viable tdTomato^hi^ single cells were re-suspended in DMEM supplemented with 10% FBS, antibiotics and antifungals, diluted with suspension reagent for attribution of optimal buoyancy, and loaded onto a large 17–25 µm 96-well microfluidic IFC (Fluidigm) for single cell capture in the automated C1 system for single-cell genomics (Fluidigm). Capture efficiency was assessed using bright field and fluorescent microscopy. Only cells captured singly (singlets) per micro-well were considered for downstream analyses. Double (doublets) and multiple (multiplets) cells captured per well were excluded. Lysis, RT, and cDNA pre-amplification were performed in loco (protocol 1.773×) with ultra-low input RNA reagents as suggested per manufacturer (Clontech). RNA spike-in controls were omitted. cDNA concentrations were estimated using Qubit 2.0 (Thermo) and cDNAs with concentration ≥ 1.0 ng/μl were used for downstream library preparation. Libraries were amplified using the Nextera XT v2 Index Kit (Illumina). Quality control on multiplexed libraries was performed using the Agilent Bioanalyzer and quantification was performed using KAPA for Illumina Sequencing Platforms (Illumina). Multiplexed libraries were sequenced as paired-end on an Illumina Next-Seq500 platform (Illumina).

### Full length transcript alignment and quantification

Demultiplexed, paired-end FASTQs were aligned to the mouse genome (mm10/gencode.Mv13) using Bowtie (version 1.0.0) with the following standard parameters: rsem-prepare-reference –bowtie –gtf and quantified using the RNA-seq by Expectation-Maximization algorithm (RSEM) (version 1.2.31) with the following standard parameters: rsem-calculate-expression -p $CORES –paired-end. Samples displaying ≥ 159,000 aligned reads were considered for downstream quality control filtering.

### Quality control metrics for full length transcripts

Cells with a total number of expressed genes > 11,000, a proportion of counts in mitochondrial genes > 15%, and genes expressed in < 3 cells were excluded for downstream analyses. Fifty-six cells were removed from all replicates, leading to a total of 288 cells (116 cells at 12 days PW, 100 cells at 15 days PW, and 72 cells at 21 days PW) for downstream analyses. To adjust systematic variation in the relationship between transcript-specific expression and sequencing depth, we normalized our data using the quantile regression-based method SCnorm^70^. Samples were then natural log-transformed and added a pseudo-count of 1 followed by batch correction for each time point using the Bayesian-based method ComBat using the ComBat function from the sva R package. The batch corrected data were used for downstream analyses.

### Single-cell western blot

Single-cell western blot assays were performed using the ProteinSimple Milo platform with the standard scWest Kit as per manufacturer’s protocol. scWest chips were rehydrated and loaded with cells at a concentration of 1 × 10^5^ cells in 1 mL suspension buffer. Doublet/multiplet capture rate in scWest chip microwells was determined with light microscopy (~1.3%, established from > 1000 microwells). scWest chips were lysed for 10 s and immediately followed by electrophoresis for 80 s at 240 V. Protein immobilization was achieved with UV light exposure for a total of 4 min. scWest chips were sequentially probed with primary and secondary antibodies for 1 h. Primary antibodies used were goat anti-mCherry (1:10, SicGen) and rabbit anti-LYZ (1:20, Abcam). Secondary antibodies used were donkey anti-goat Alexa Fluor 647 (1:40, Thermo) and donkey anti-rabbit Alexa Fluor 555 (1:40, Thermo). Slides were washed, centrifuge-dried, and imaged with the GenePix 4000B Microarray Scanner (Molecular Devices). Data was analyzed using Scout Software (ProteinSimple) and ImageJ (NIH). Debris, artifacts, and false positive signals were manually excluded during data analyses.

### Reporting Summary

Further information on experimental design is available in the Nature Research Reporting Summary linked to this Article.

## Supplementary Information

Supplementary Information

Description of Additional Supplementary Files

Supplementary Data 1

Supplementary Data 2

Supplementary Data 3

Supplementary Data 4

Supplementary Data 5

Supplementary Data 6

Reporting Summary

## Data Availability

The authors declare that all supporting data are available within the Article and its Supplementary Information files. 3′-end scRNA-seq data have been deposited in the GEO database under accession code GSE113854. Full length scRNA-seq data have been deposited in the GEO database under accession code GSE113605.
