# Supplementary material for: Single-cell analysis reveals fibroblast heterogeneity and myeloid-derived adipocyte progenitors in murine skin wounds
Source: Nat Commun. 2019 Feb 8;10:650. doi: 10.1038/s41467-018-08247-x (PMC6368572; doi:10.1038/s41467-018-08247-x)
Supplement: Supplementary file 1 — Supplementary Information [file 41467_2018_8247_MOESM1_ESM.pdf]

Supplementary Information

Single-cell analysis reveals fibroblast heterogeneity and myeloid-derived adipocyte progenitors in murine skin wounds

Christian F. Guerrero-Juarez et al.

Supplementary Figures

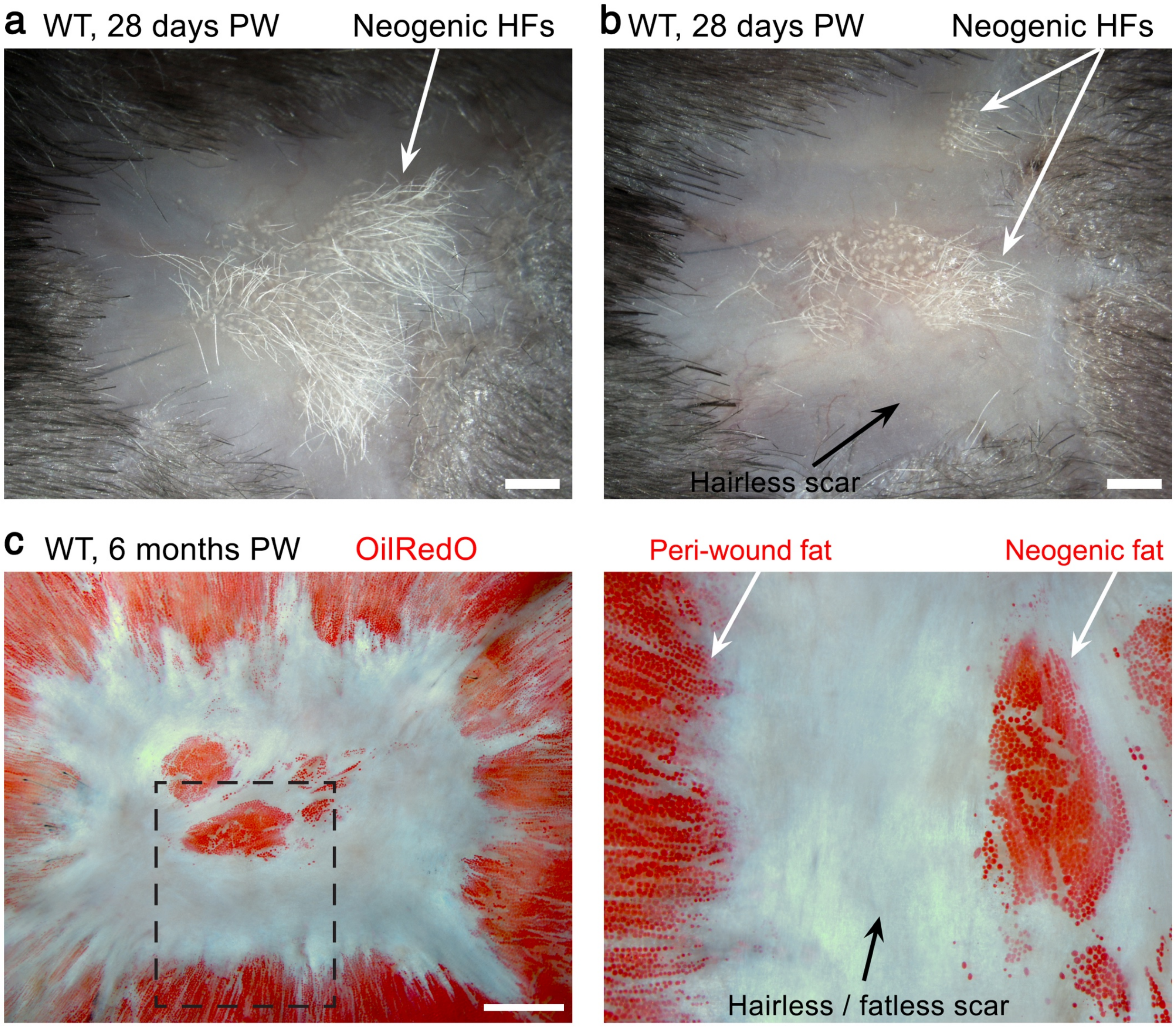

Supplementary Figure 1. Wound induced regeneration of hair follicles and fat. (a, b) Large full-thickness excisional wounds in adult wild type mice regenerate de novo hair follicles in their center. Representative wound samples from 28 days post-wounding are shown. **(c)** De novo adipocytes regenerate around new hair follicles in the center of large wounds. Representative wound sample from 6 months post-wounding is shown. Adipocytes are stained red with OilRedO. PW, post-wounding; HF, hair follicle; WT, wild type. Size bars: a-c – 1 mm.

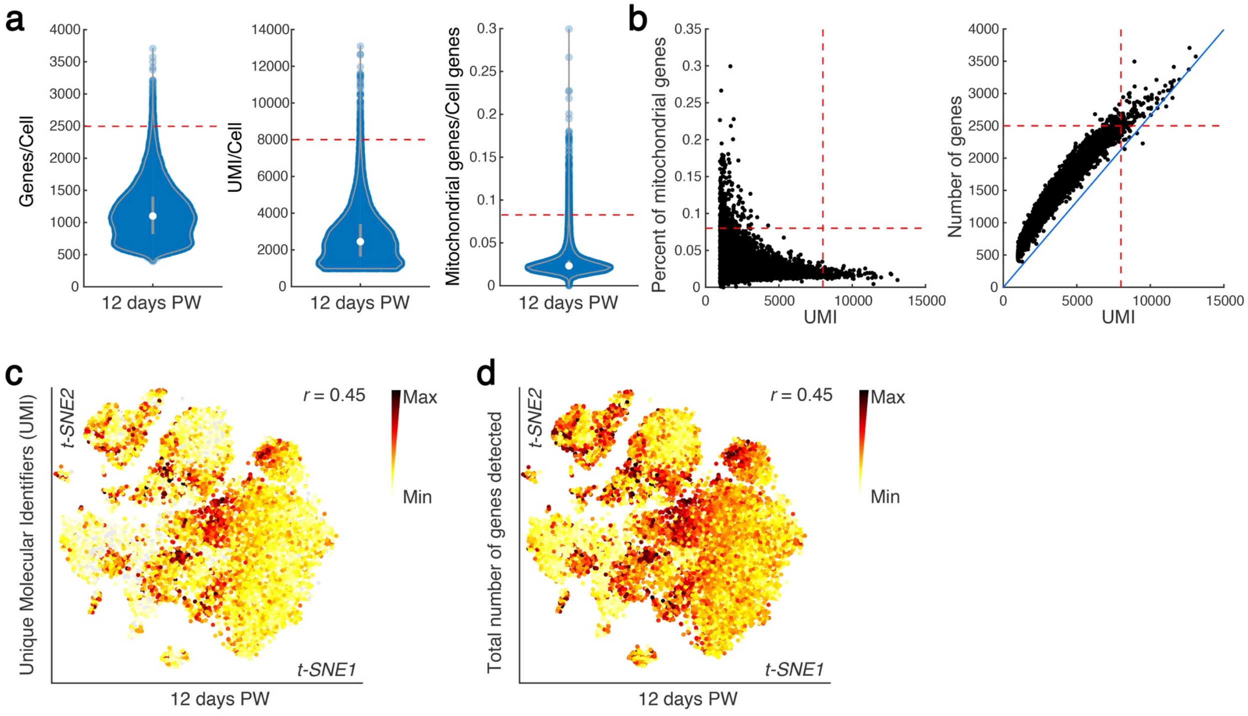

Supplementary Figure 2. Quality control metrics of scRNA-seq data from day 12 wounds. (a, b) Genes/Cell, Unique Molecular Identifiers (UMI)/Cell, and percent of mitochondrial genes/cell genes are shown. Red lines on **(a)** mark cut-offs for selecting cells for downstream analyses. **(c)** t-SNE plot with color-coded UMIs per cell is shown. Cells with the highest UMI are colored black. **(d)** t-SNE plot with color-coded number of expressed genes per cell is shown. Cells with the highest number of expressed genes are colored black. PW, post-wounding day.

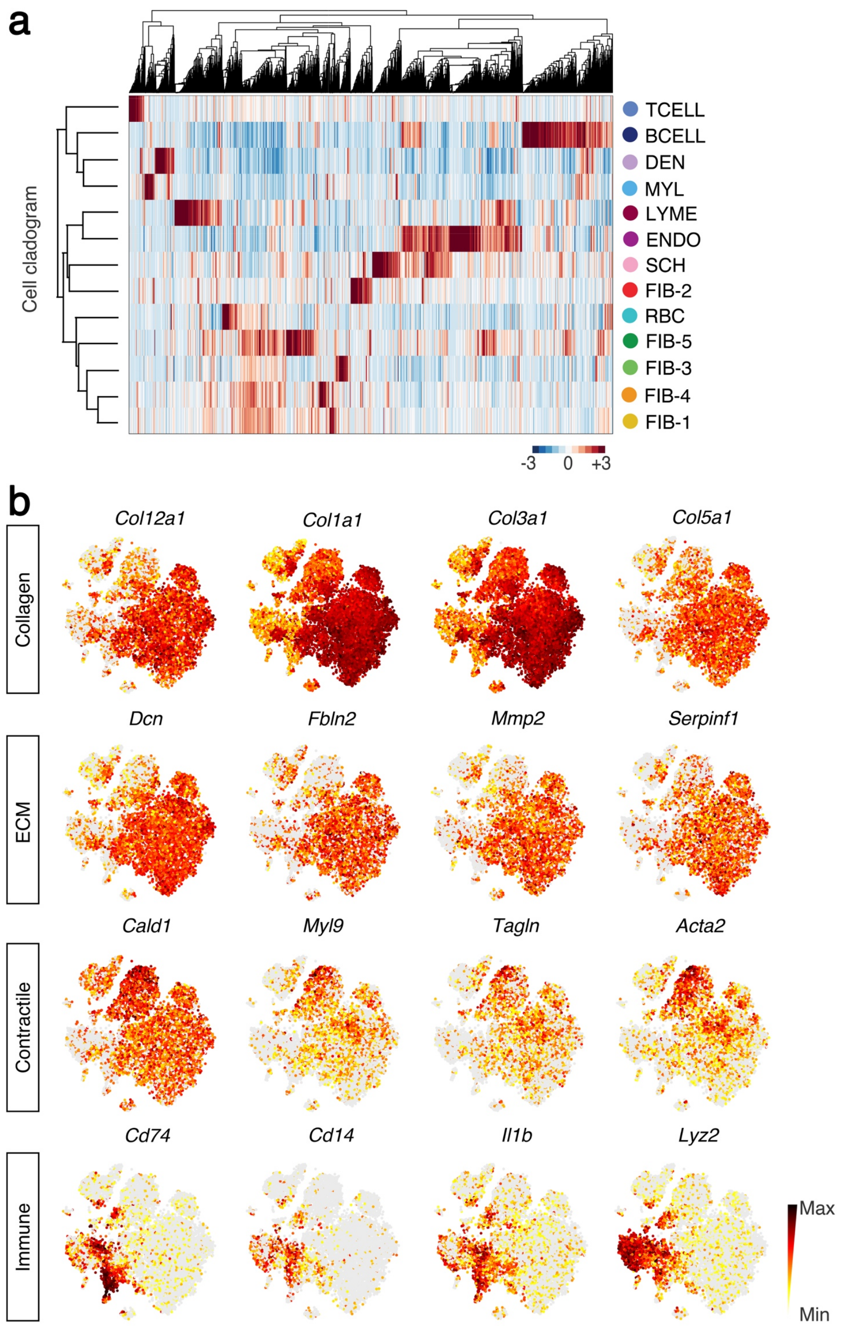

Supplementary Figure 3. Expression of selected genes across wound cell populations. (a) Unsupervised hierarchical clustering of all wound cell clusters based on the average expression of highly variable genes (correlation distance metric, average linkage). **(b)** Feature plots showing expression of selected genes. Top row shows collagen genes, second – extracellular matrix (ECM) genes, third – genes encoding for contractile proteins, and fourth – genes encoding for immune markers. Expression levels for each cell are color-coded and overlaid onto t-SNE space. Cells with the highest gene expression levels are colored black.

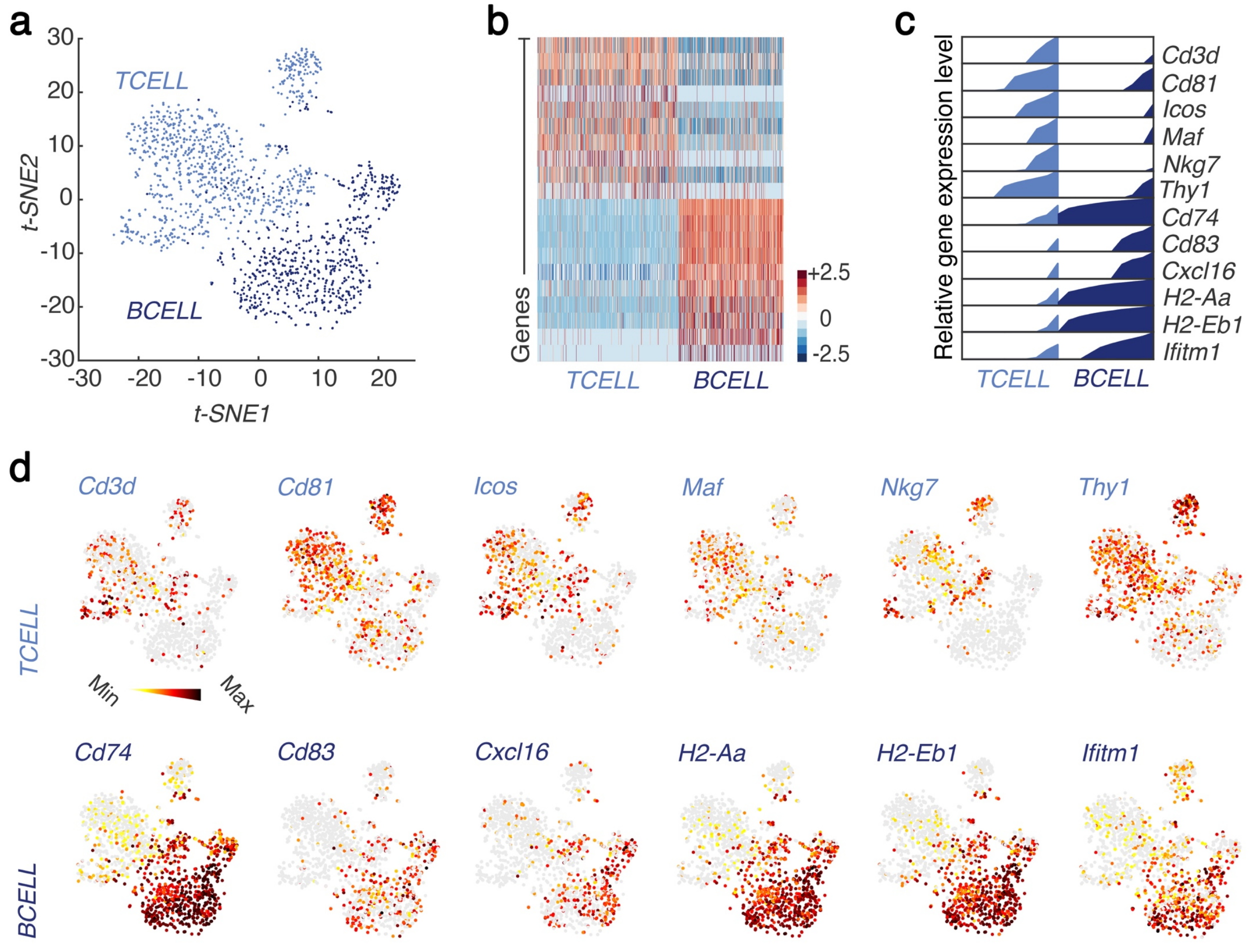

Supplementary Figure 4. Identification of lymphoid cell types in day 12 wounds. (a) t-SNE plot showing clustering of T lymphocytes (TCELL, light blue) and B lymphocytes (BCELL, dark blue). **(b)** Heatmap of differentially expressed genes between TCELL and BCELL clusters. **(c, d)** Differential expression of marker genes shown as bar charts **(c)** and feature plots **(d)**. Expression levels for each cell on **(d)** are color-coded and overlaid onto t-SNE space. Cells with the highest gene expression levels are colored black.

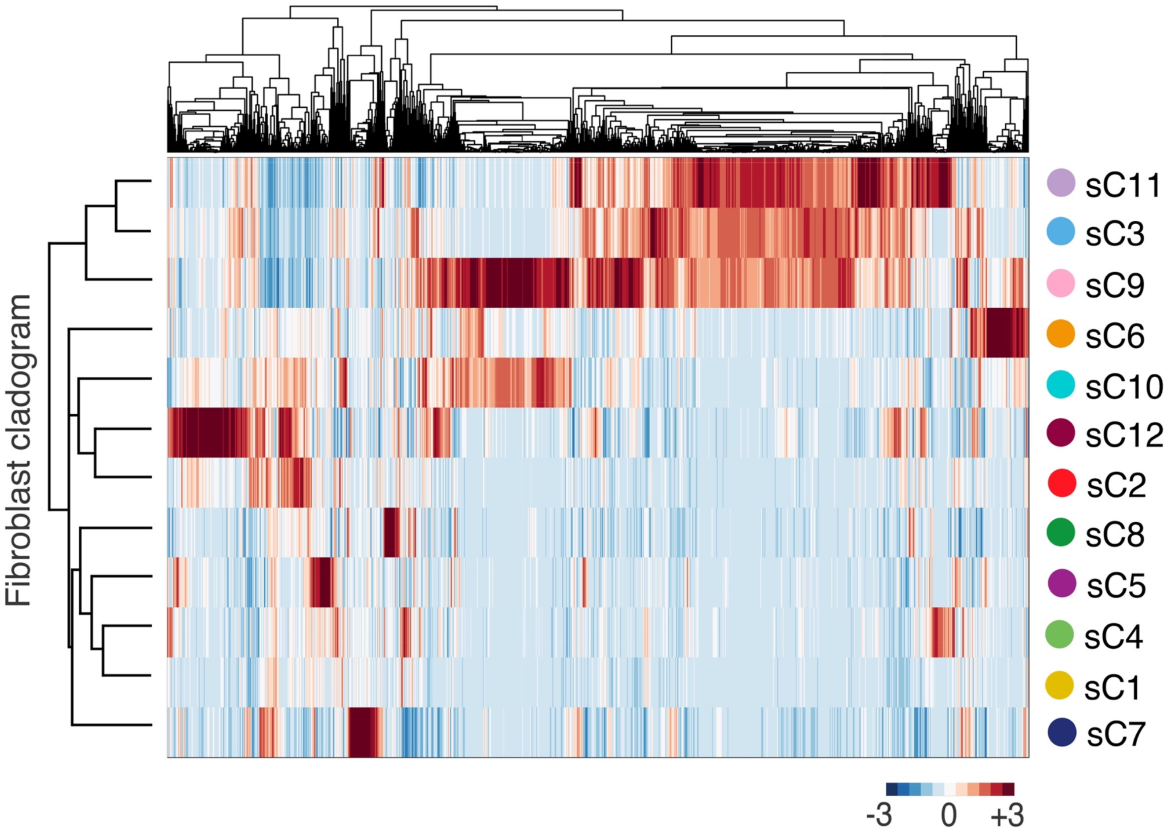

Supplementary Figure 5. Unsupervised hierarchical clustering analysis on day 12 wound fibroblasts. Hierarchical clustering on all fibroblasts based on the average expression of highly variable genes (correlation distance metric, average linkage).

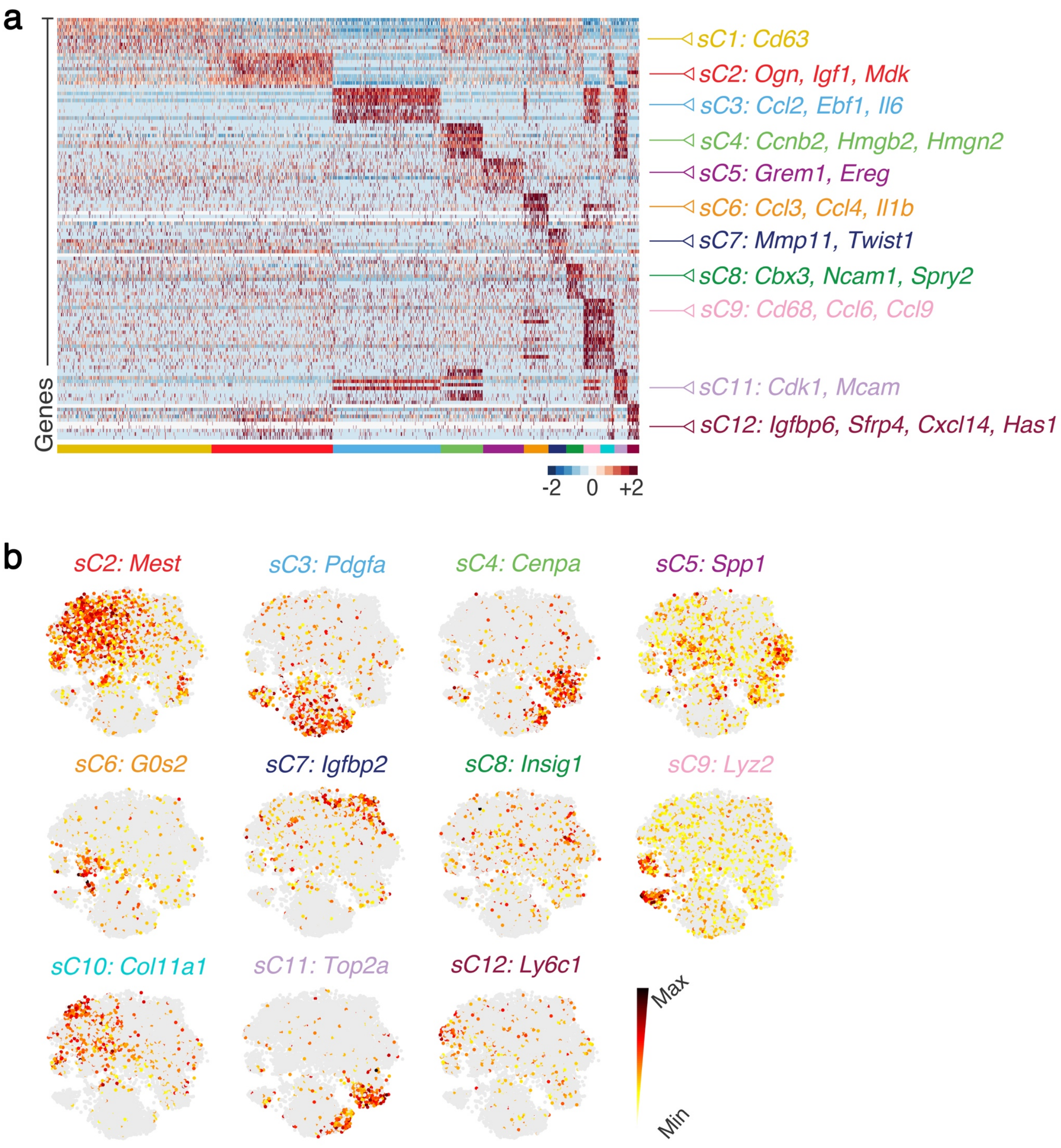

Supplementary Figure 6. Identification of heterogeneity among wound fibroblasts. (a) Heatmap of differentially expressed genes between wound fibroblasts with selected marker genes (on the right). **(b)** Feature plots of selected subcluster-specific marker genes. Expression levels for each cell are color-coded and overlaid onto t-SNE space. Cells with the highest gene expression levels are colored black.

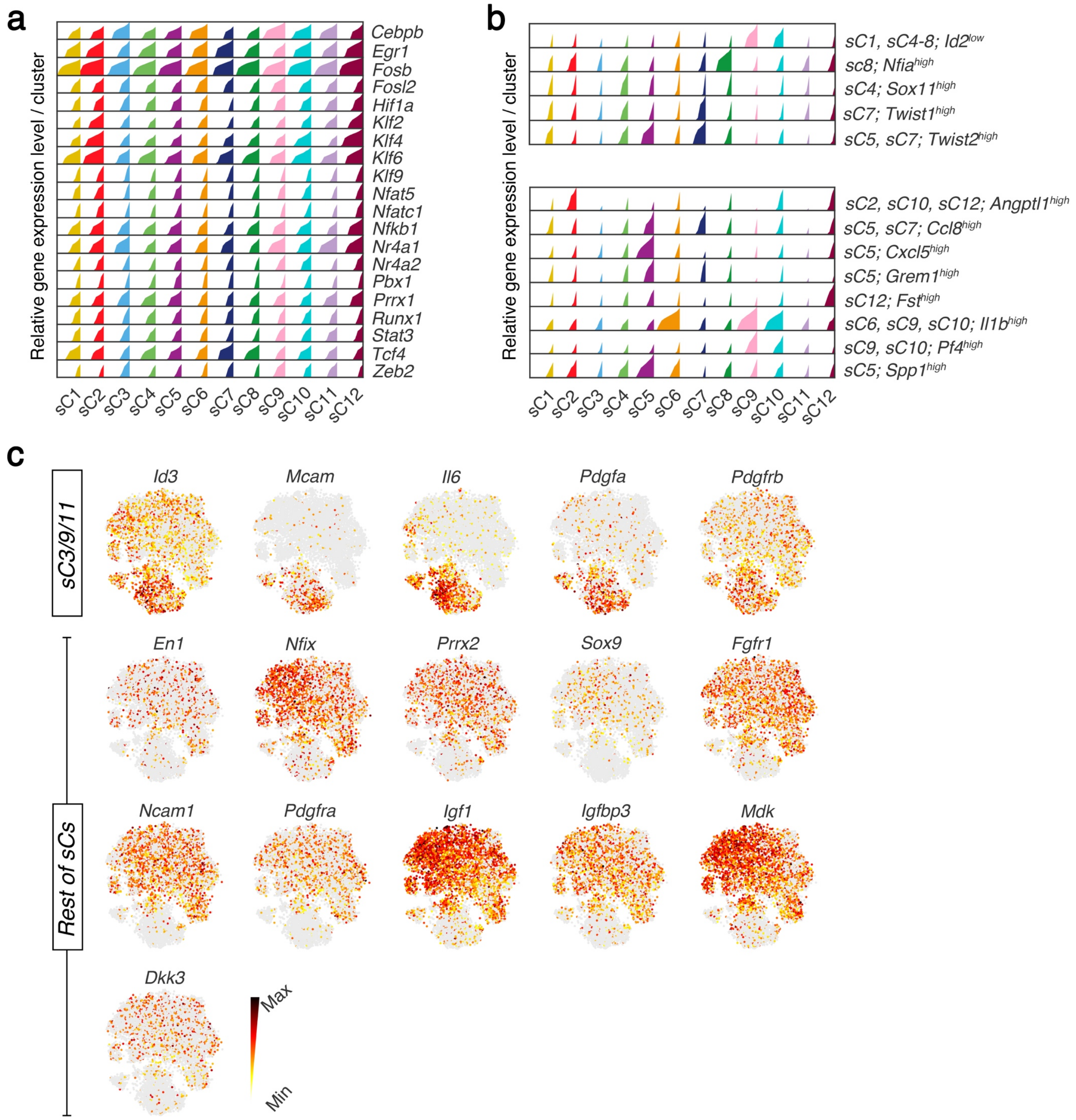

Supplementary Figure 7. Identification of shared and unique marker genes in day 12 wound fibroblast subclusters. (a) Bar charts showing expression levels for common wound fibroblast transcription factors across all fibroblast subclusters. **(b)** Bar charts showing expression levels for subcluster-specific transcription factors and secreted signaling factors. **(c)** Feature plots of differentially expressed marker genes between fibroblast subclusters sC3/9/11 and the rest of fibroblasts. Expression levels for each cell are color-coded and overlaid onto t-SNE space. Cells with the highest gene expression levels are colored black.

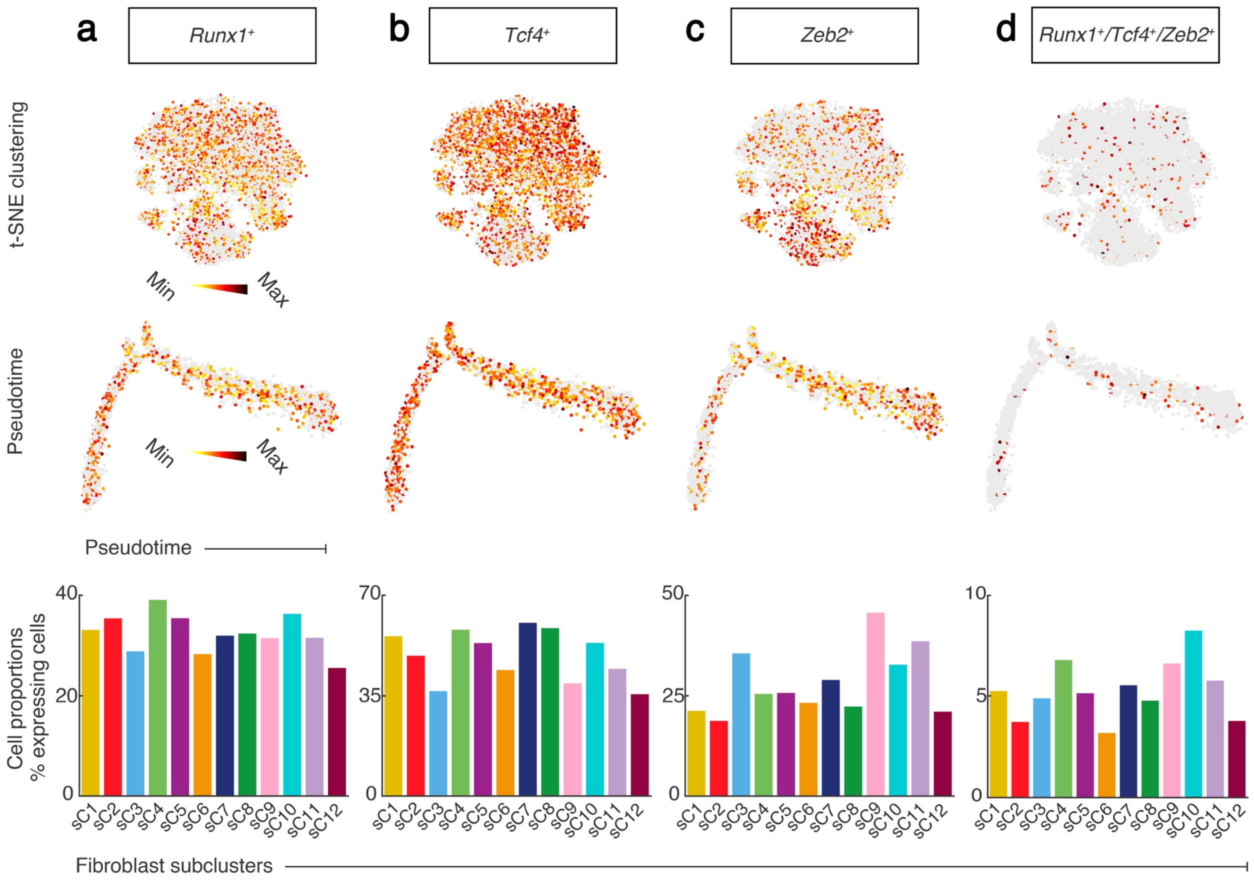

Supplementary Figure 8. Expression of pro-contractile transcription factors across all day 12 wound fibroblasts. Expression patterns for transcription factors *Runx1* **(a)**, *Tcf4* **(b)** and *Zeb2* **(c)** are shown. For each gene, feature plot overlays onto t-SNE space (top panels), pseudotime (middle panels) as well as bar charts (bottom panels) are shown. **(d)** Distribution of wound fibroblasts co-expressing *Runx1, Tcf4* and *Zeb2*. Small percentage of wound fibroblasts across all subclusters was *Runx1/Tcf4/Zeb2* triple-positive.

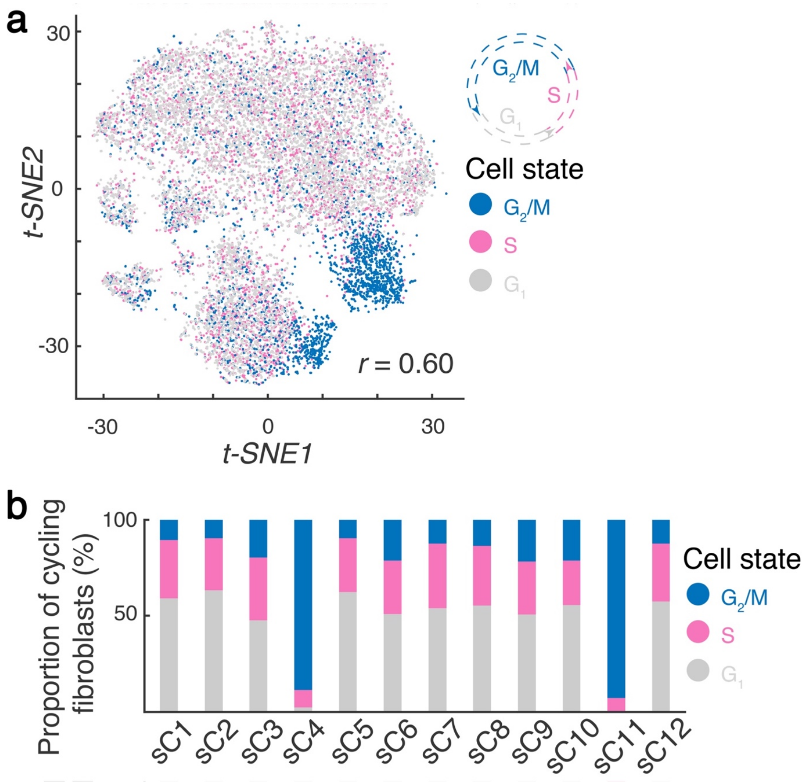

Supplementary Figure 9. Cell cycle metrics on day 12 wound fibroblasts. (a) t-SNE plot of assigned cell states on day 12 wound fibroblasts. Cells in S phase are colored pink, G2/M phase – blue and G1 phase – grey. **(b)** Proportion of cell cycle stages per fibroblast subcluster.

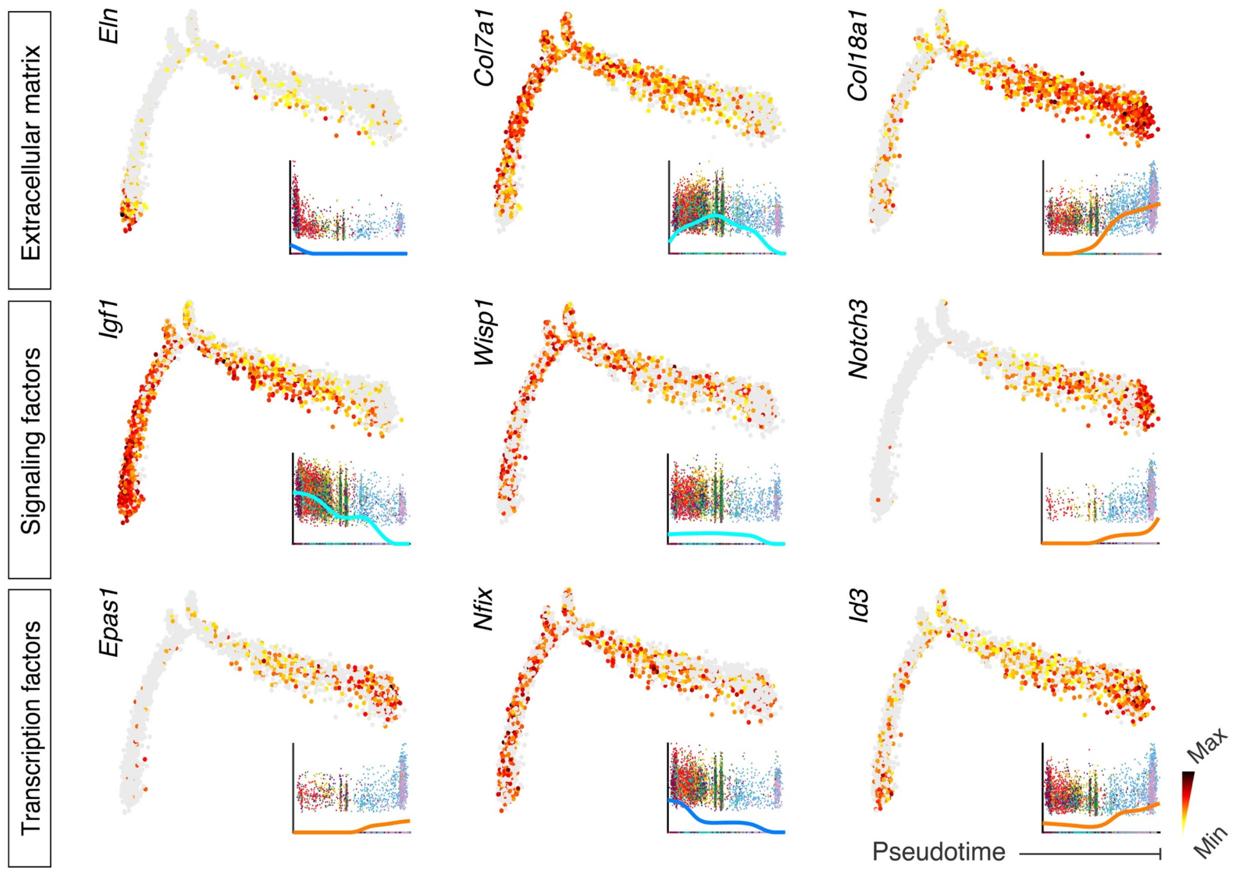

Supplementary Figure 10. Pseudotime analysis on fibroblasts from day 12 wounds. Selected genes encoding for extracellular matrix molecules (top row), signaling factors (middle row) and transcription factors (bottom row) are shown. Expression levels for each cell are color-coded and overlaid onto pseudotime. Cells with the highest gene expression levels are colored black. Genes were selected as highly expressed in cells either at the beginning of RNA velocity paths Path1 and Path2 – *Eln, Igf1, Nfix*; at the end of Path1 and Path2 – *Col7a1, Wisp1*; or at the end of Path3 – *Col18a1, Notch3, Id3* and *Epas1*.

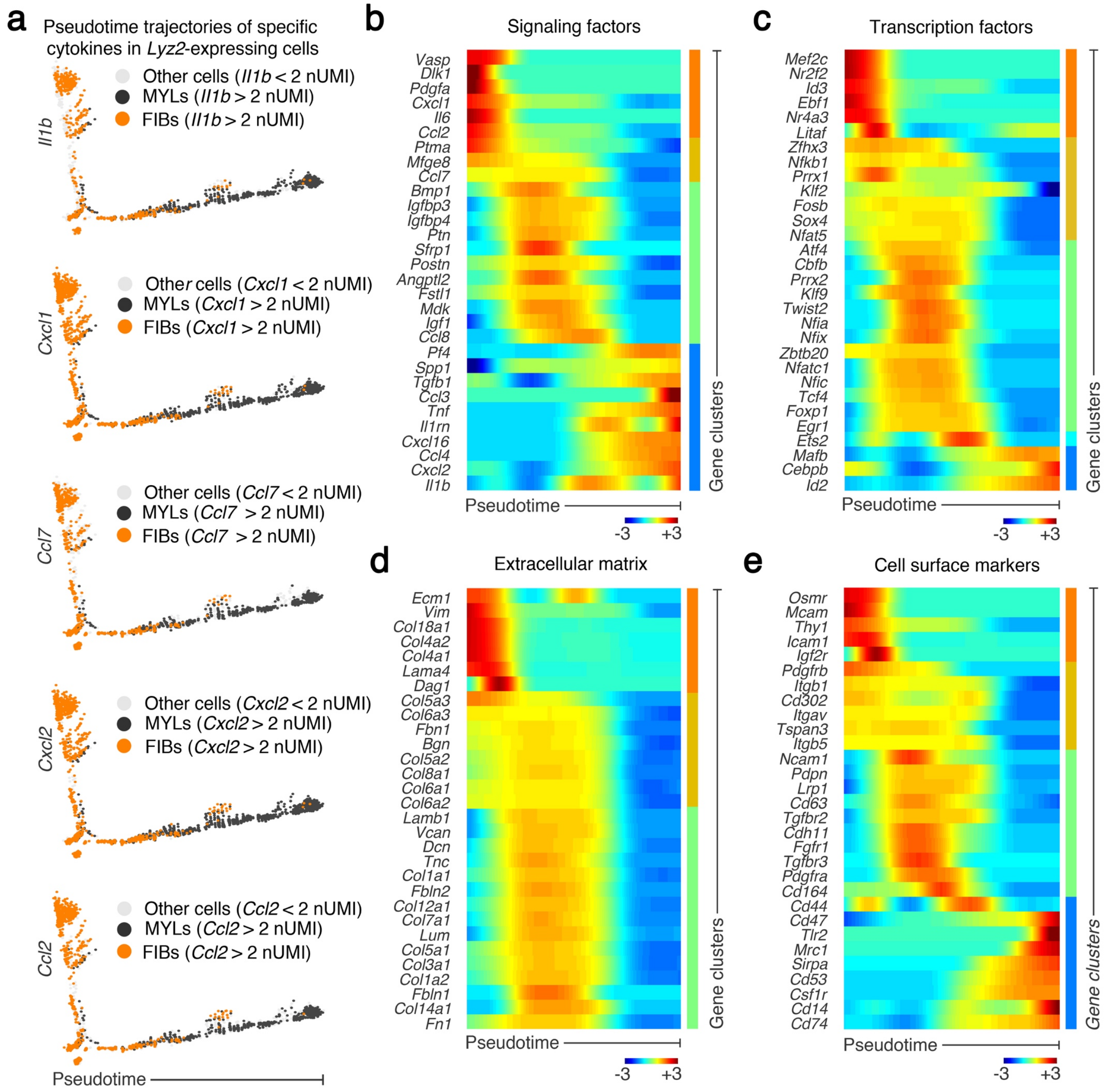

Supplementary Figure 11. Gene expression dynamics across the pseudotime for *Lyz2*$^{+}$ myeloid cells and wound fibroblasts. (a) Distribution of cells expressing cytokine markers shown as pseudotime feature plots. Cells are colored by either orange or black if their normalized UMIs (nUMIs) are > 2. Positive fibroblasts are colored orange, positive myeloid cells – black and negative cells – light grey (nUMIs < 2). Positive cells are present across the entire pseudotime. **(b-e)** Rolling wave plots for selected signaling factors **(b)**, transcription factors **(c)**, extracellular matrix molecules **(d)** and cell surface markers **(e)** across *Lyz2*$^{+}$ fibroblast and myeloid cell pseudotime trajectory. FIB, fibroblast; MYL, myeloid cell.

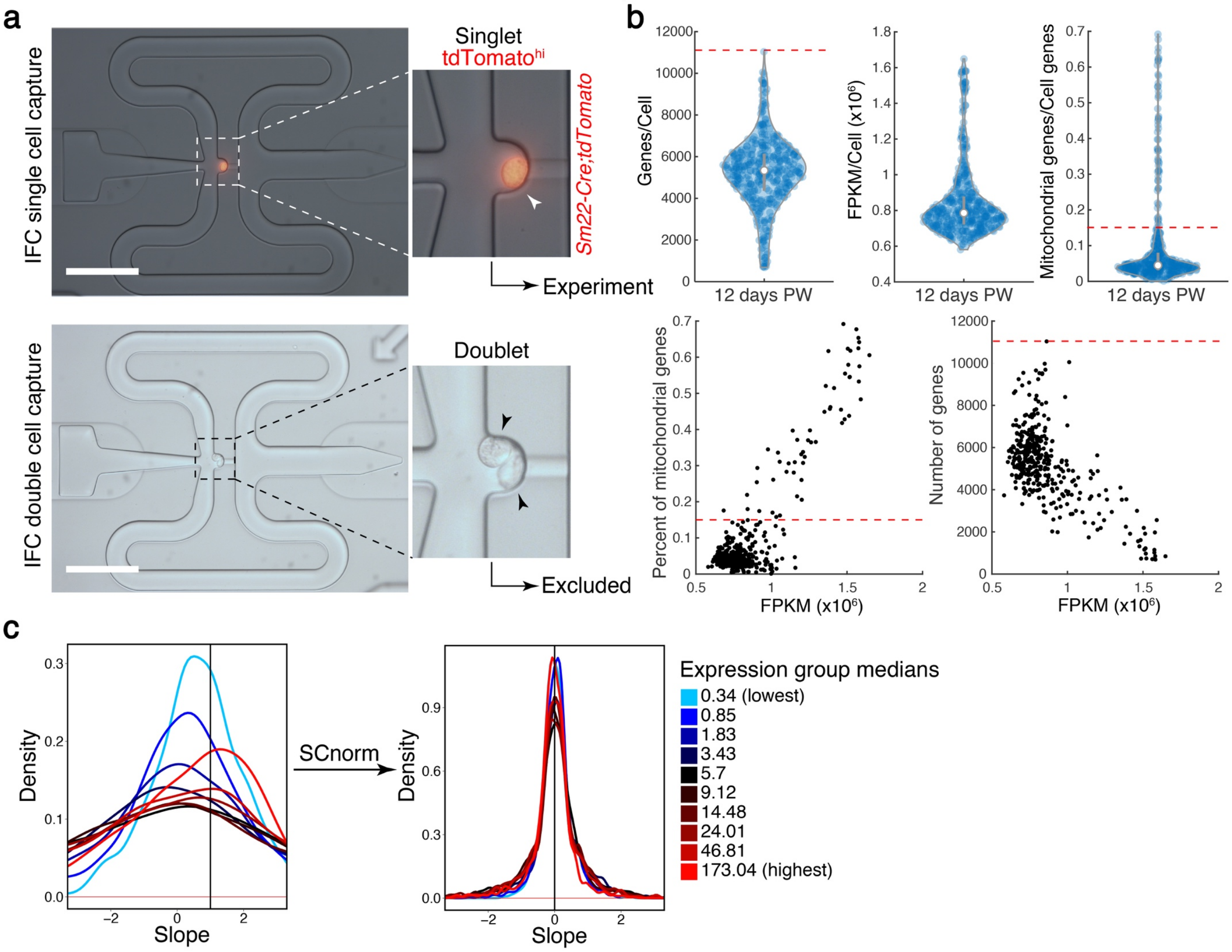

Supplementary Figure 12. Microfluidic capture efficiency and quality control of full-length scRNA-seq data from day 12 wounds. (a) Visual discrimination of automated singlet and doublet cell capture is shown. **(b)** Genes/Cell, FPKM/Cell, and percent of mitochondrial genes/cell genes are shown. Red lines mark upper cut-offs for selecting cells for downstream analyses. **(c)** scRNA-seq data normalization procedure. Graph on the left shows pre-normalization distribution of gene expression counts to sequencing depth for ten equally sized groups of genes. Each gene group is color-coded. Graph on the right shows post-normalization count-depth relationship. IFC, integrated fluidic circuit; PW, post-wounding.

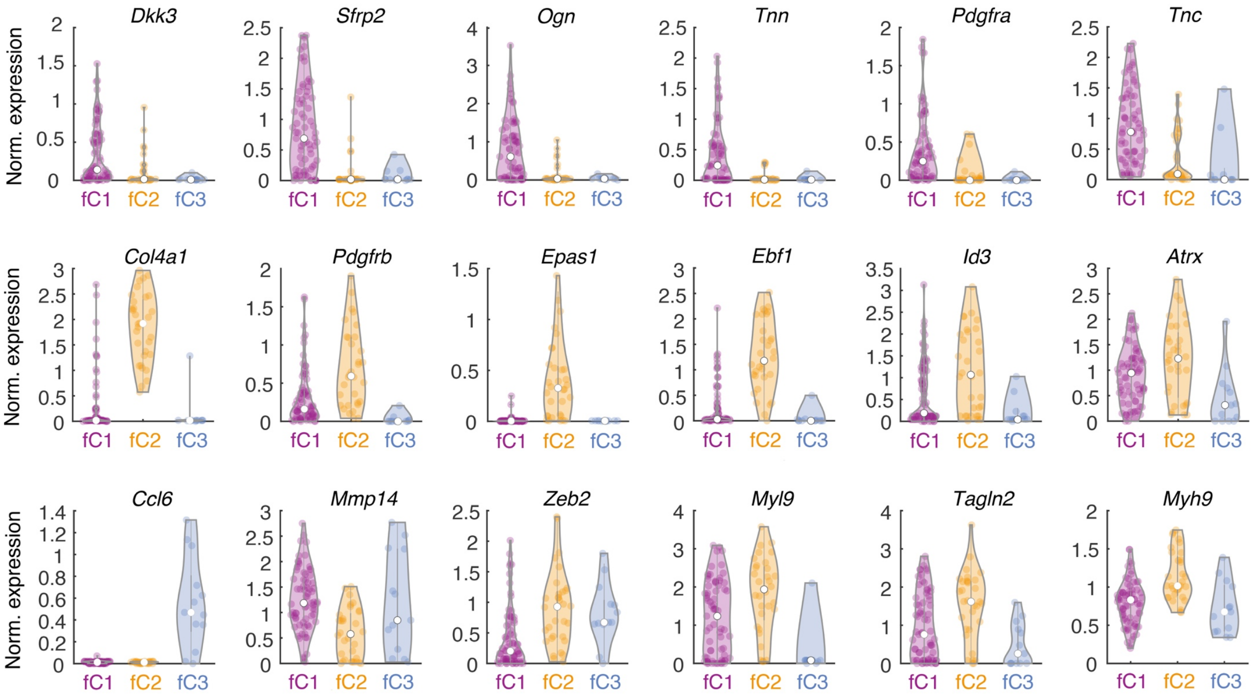

Supplementary Figure 13. Expression of selected genes in contractile fibroblasts from day 12 wounds. Violin plots showing expression of selected genes in three clusters of sorted tdTomato^{hi} contractile cells, fC1 through fC3, from day 12 wounds. Expression in clusters is color-coded. Top row shows genes highly expressed in fC1 cluster, middle row – genes in fC2 cluster. Bottom row shows genes highly expressed in fC3 cluster (*Ccl6*) or genes shared between clusters (*Mmp14* through *Myh9*).

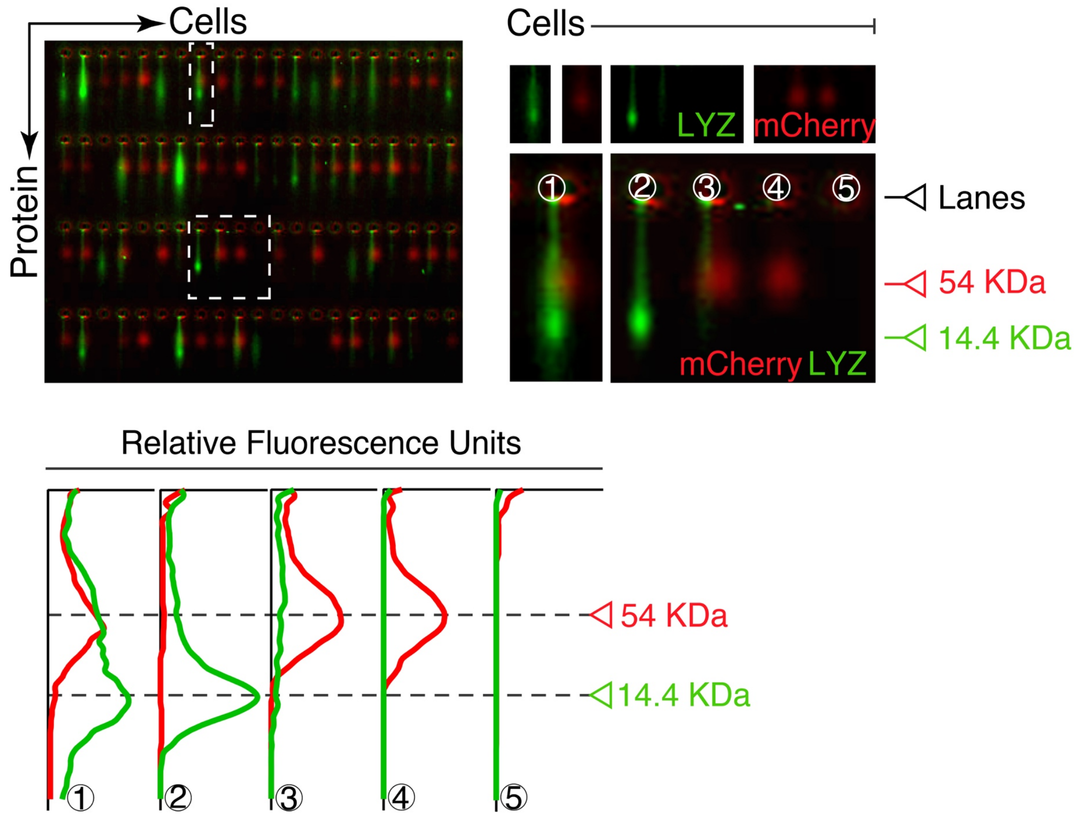

Supplementary Figure 14. Single-cell western blot reveals LYZ-expressing wound myofibroblasts. Single-cell western blot on unsorted cells isolated from day 12 *Sm22-Cre;tdTomato* wounds is shown. Co-staining for LYZ (green) and tdTomato (mCherry, red) reveals double positive cells (lane #1), LYZ single-positive cells (lane #2), tdTomato single-positive cells (lanes #3, #4) and double negative cells (lane #5). Graph on the bottom shows relative fluorescence for selected lanes.

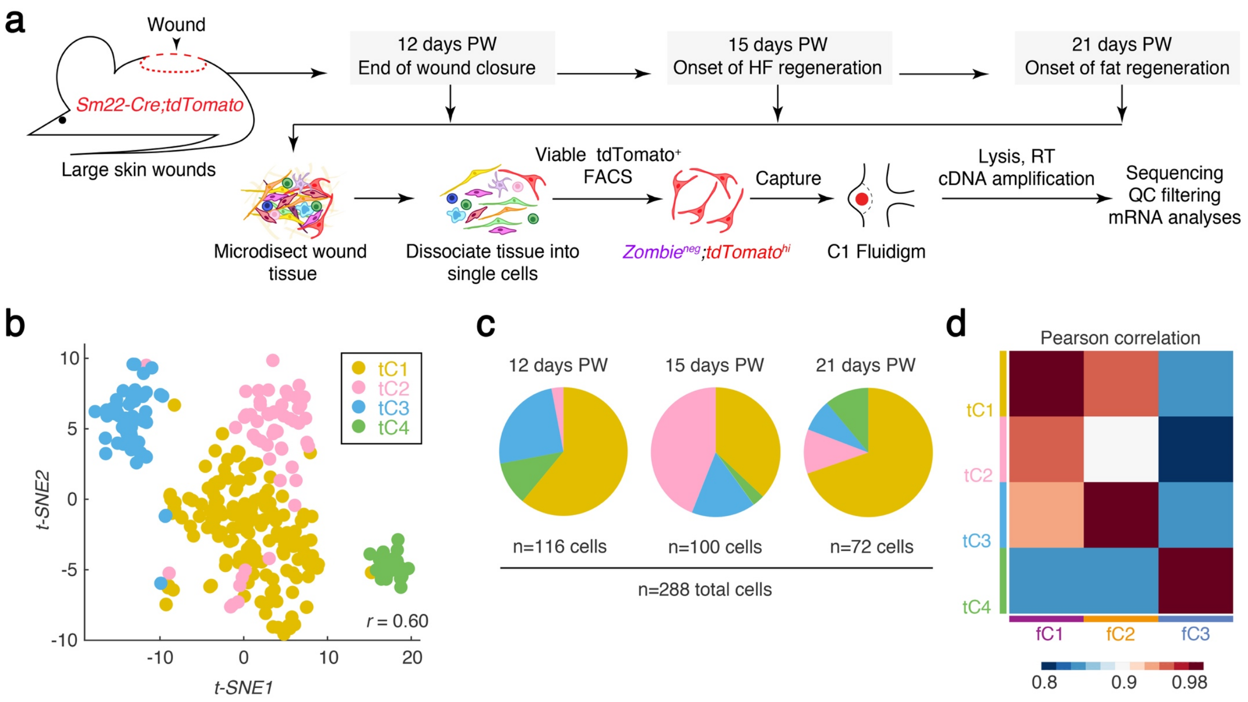

Supplementary Figure 15. Distinct fibroblast states persist in large skin wounds. (a) Schematic of the isolation strategy of tdTomato^{hi} contractile cells from wounds of *Sm22-Cre;tdTomato* mice at specified post-wounding time points. Cells were isolated for analysis at 12 days PW – completion of wound closure, 15 days PW – onset of hair follicle regeneration, and 21 days PW – onset of fat regeneration. **(b)** t-SNE plot of tdTomato^{hi} cells from all three post-wounding time points is shown. Total of 116 cells were analyzed on 12 days PW, 100 cells on 15 days PW, and 72 cells on 21 days PW. All cells group into four distinct clusters, tC1 through tC4. Cells from distinct clusters are color-coded. **(c)** Wounds from each post-wounding time point contain cells from all four clusters, albeit at different ratios. **(d)** Pearson correlation heatmap between three tdTomato^{hi} cell clusters from 12 days PW (fC1, fC2 and fC3) and four tdTomato^{hi} cell clusters formed by combined cells from 12, 15 and 21 days PW (tC1, tC2, tC3 and tC4). PW, post-wounding; HF, hair follicle; FACS, fluorescence activated cell sorting; RT, reverse transcription; QC, quality control.

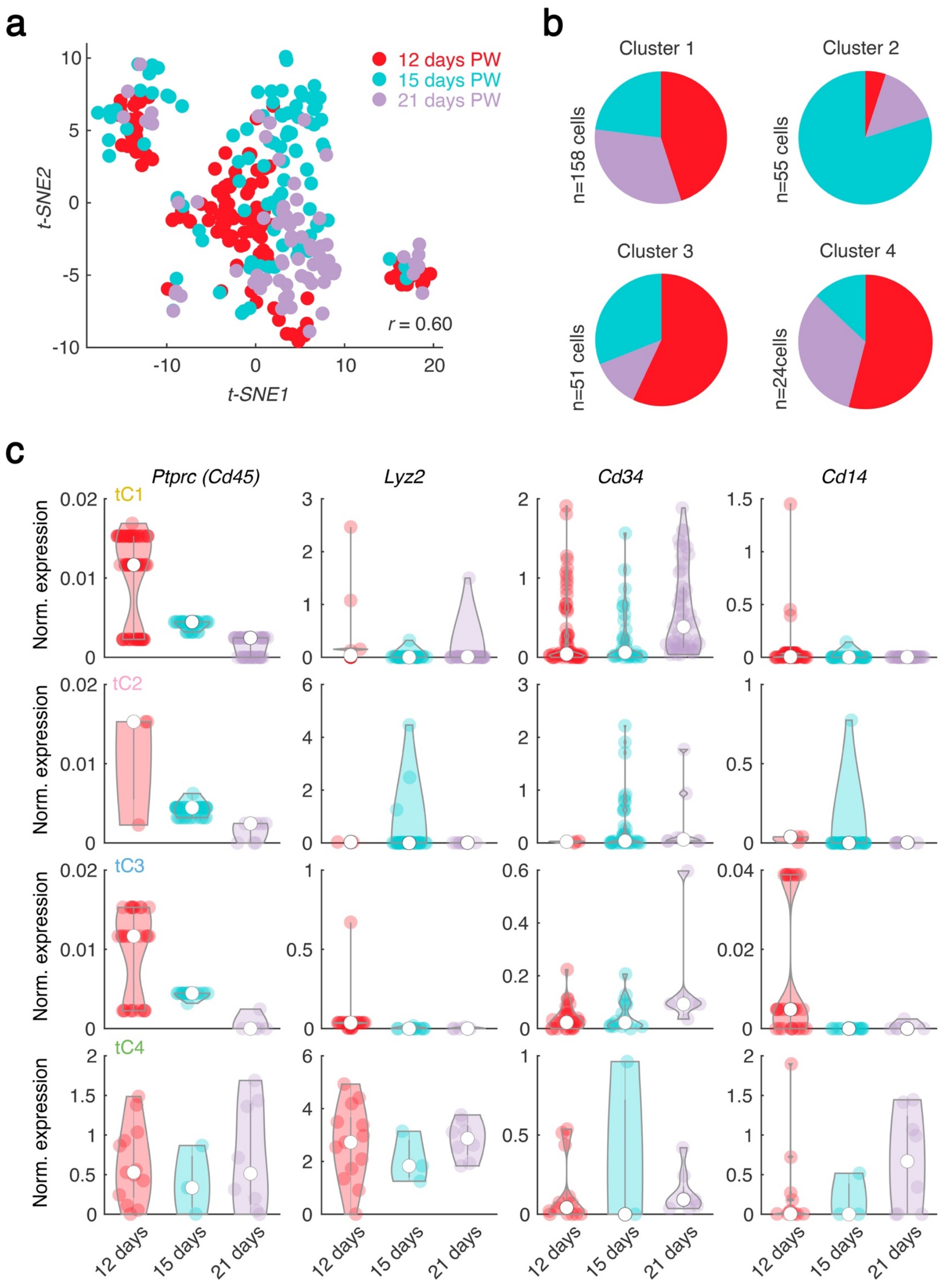

Supplementary Figure 16. Myeloid marker-expressing contractile fibroblasts persist in large wounds. (a) t-SNE plot color-coded by post-wounding time point. **(b)** Each cluster contains cells from all three post-wounding time points. **(c)** Violin plots of selected genes in four cell clusters, showing persistence of myeloid marker-expressing cells in cluster tC4 across all three time points, 12, 15 and 21 days PW. PW, post-wounding.

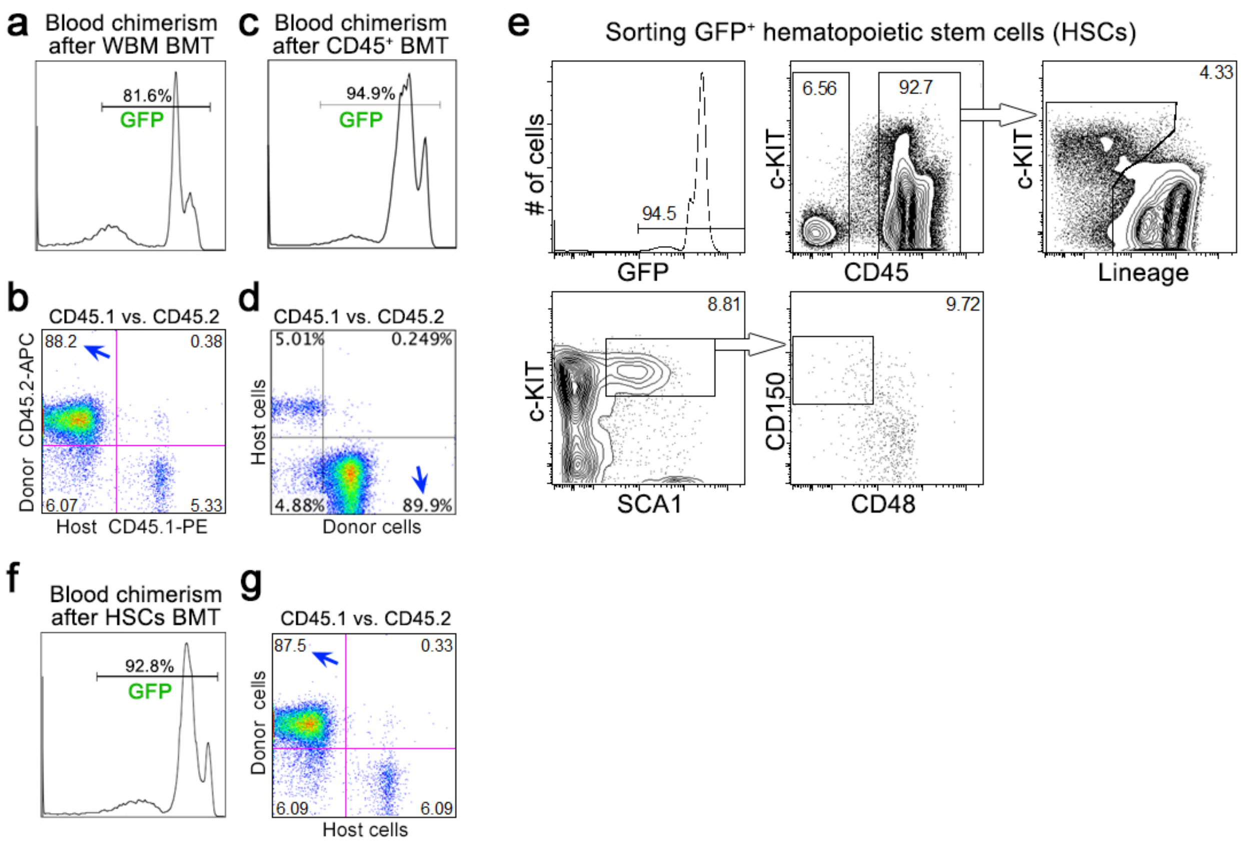

Supplementary Figure 17. Generation of the bone marrow transplantation mouse models. (a-d, f, g) Bone marrow reconstitution levels in all BMT mice were validated by FACS of the peripheral blood for either GFP **(a, c, f)** or CD45.1 vs. CD45.2 **(b, d, g)**. **(e)** Details of the SLAM-based cell sorting strategy used to isolate hematopoietic stem cells. WBM, whole bone marrow; BMT, bone marrow transplantation; HSC, hematopoietic stem cell.

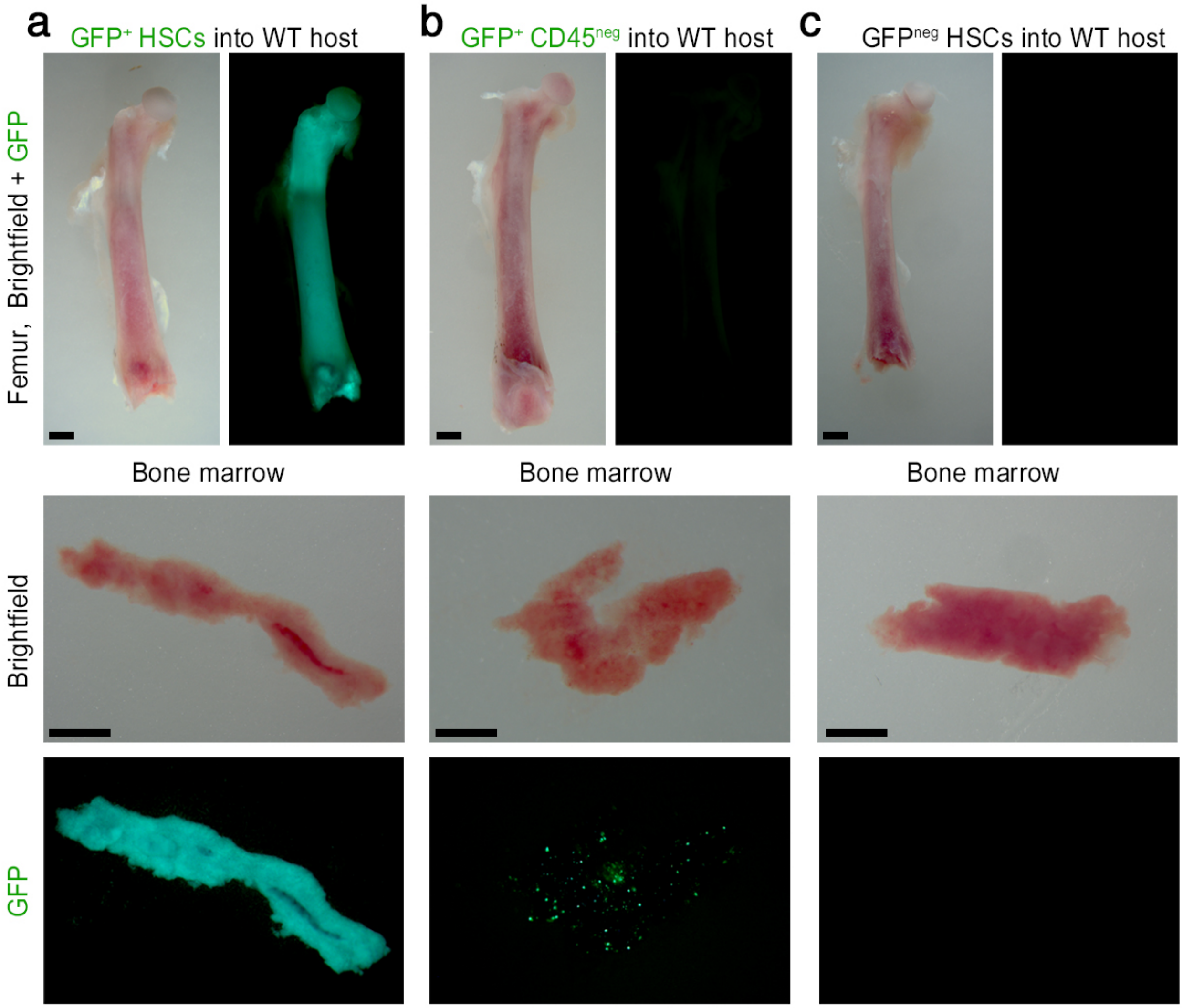

Supplementary Figure 18. Confirmation of bone marrow transplantation efficiency. (a-c) Whole mount GFP fluorescence levels of the femur (top panels) and extracted bone marrow (bottom panels) in BMT mice that were reconstituted with GFP^{+} HSCs **(a)**, GFP^{+} $CD45^{neg}$ cells **(b)** and control GFP^{neg} HSCs **(c)**. Images are representative of the GFP expression patterns observed in three or more independent samples. WT, wild type; HSC, hematopoietic stem cell. Size bars: 1mm.

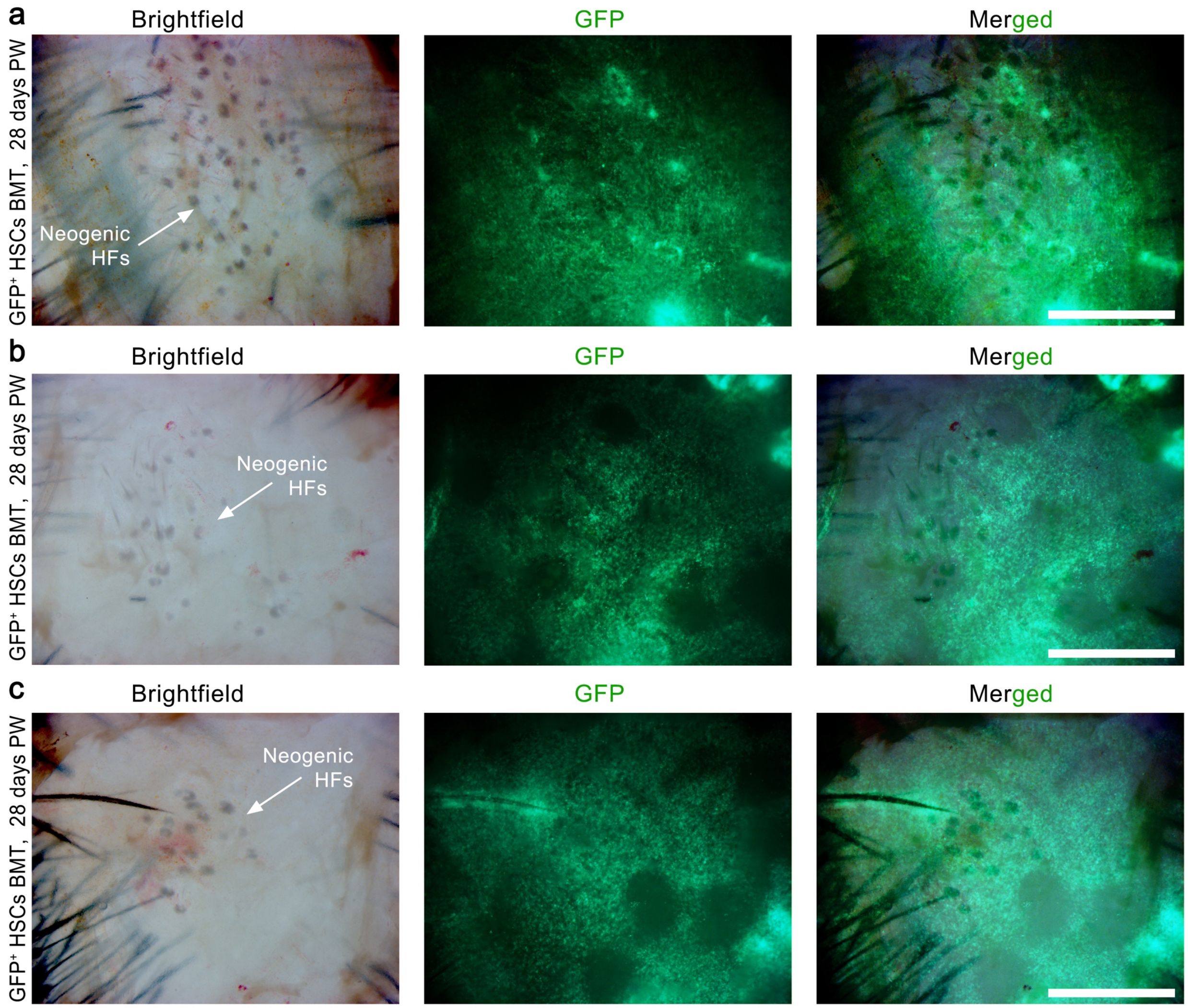

Supplementary Figure 19. Contribution of hematopoietic lineage cells toward regenerating wounds in BMT mice. (a-c) GFP^+ hematopoietic lineage cells significantly contribute to regenerating wounds on day 28 PW. Neogenic hair follicles (HFs) are marked. Images are representative of the GFP expression patterns observed in three or more independent samples. Size bars: 1 mm. PW, post-wounding; BMT, bone marrow transplantation; HSC, hematopoietic stem cell.

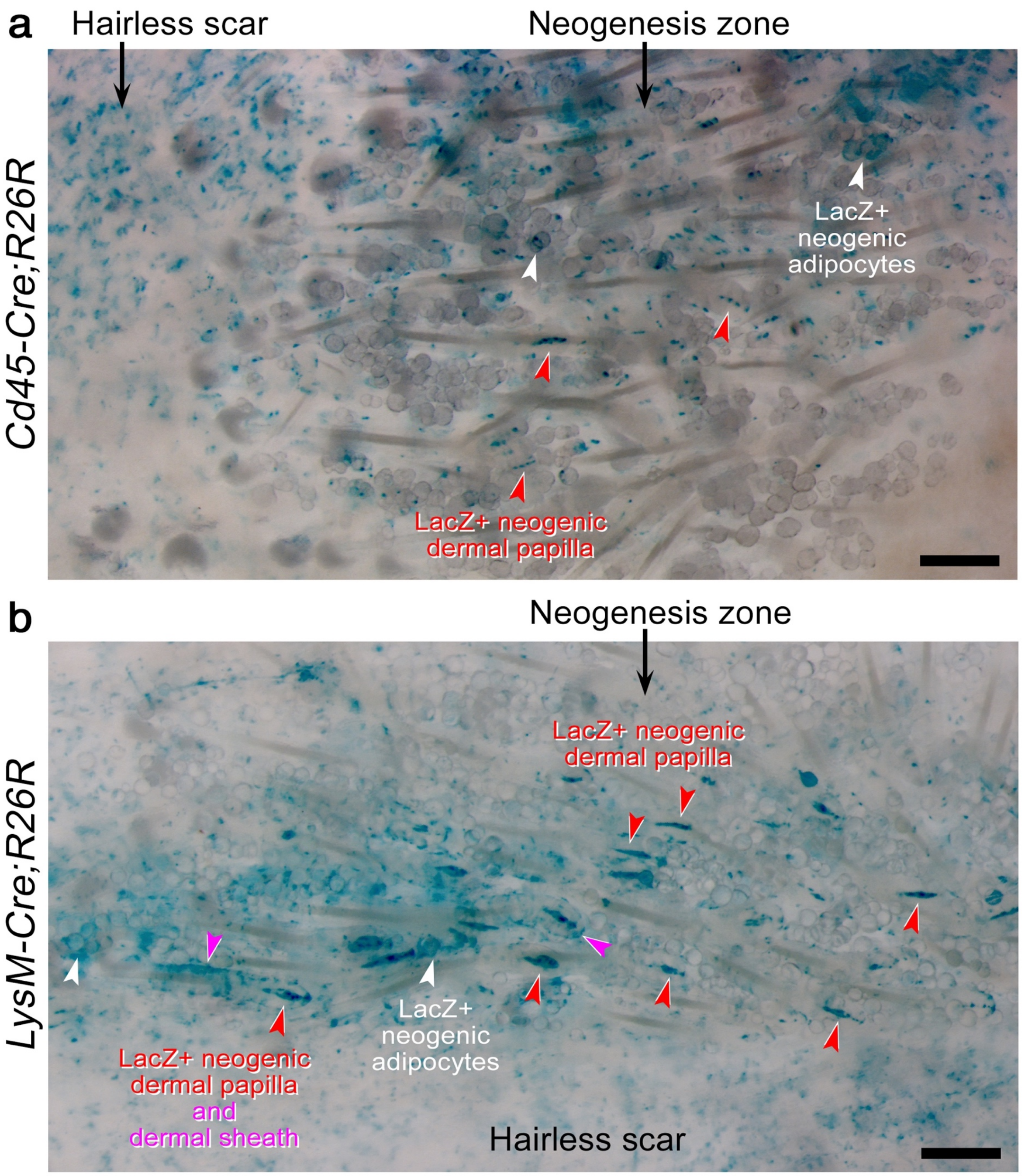

Supplementary Figure 20. Contribution of hematopoietic lineage cells toward de novo adipocytes and dermal cells of neogenic hair follicles. LacZ-positive clusters of adipocytes (white arrowheads) and lacZ-positive dermal papillae (red arrowheads) and dermal sheath of neogenic hair follicles (purple arrowheads) form in the wounds of pan-hematopoietic specific *Cd45-Cre;R26R* **(a)** and myeloid-specific *LysM-Cre;R26R* mice **(b)**. Size bars: 200 µm.
