# Supplementary material for: Single-cell analysis reveals fibroblast heterogeneity and myeloid-derived adipocyte progenitors in murine skin wounds
Source: Nat Commun. 2019 Feb 8;10:650. doi: 10.1038/s41467-018-08247-x (PMC6368572; doi:10.1038/s41467-018-08247-x)
Supplement: Supplementary file 2 — Description of Additional Supplementary Files [file 41467_2018_8247_MOESM2_ESM.docx]

**Title:** Supplementary Data 1.
**Description:** Differentially expressed gene signatures of wound cells from day 12 post-wounding.

**Title:** Supplementary Data 2.
**Description:** Differentially expressed gene signatures of wound fibroblasts from day 12 post-wounding.

**Title:** Supplementary Data 3.
**Description:** Differentially expressed genes across wound fibroblast pseudotime.

**Title:** Supplementary Data 4.
**Description:** Differentially expressed genes across fibroblast-to-myeloid pseudotime.

**Title:** Supplementary Data 5.
**Description:** Differentially expressed gene signatures of tdTomato^+^ wound cells from day 12 post-wounding.

**Title:** Supplementary Data 6.
**Description:** Differentially expressed gene signatures of tdTomato^+^ wound cells from days 12, 15 and 21 post-wounding.
