# Supplementary material for: Single-cell analysis reveals fibroblast heterogeneity and myeloid-derived adipocyte progenitors in murine skin wounds
Source: Nat Commun. 2019 Feb 8;10:650. doi: 10.1038/s41467-018-08247-x (PMC6368572; doi:10.1038/s41467-018-08247-x)
Supplement: Supplementary file 9 — Reporting Summary [file 41467_2018_8247_MOESM9_ESM.pdf]

nature research

Corresponding author(s): George Cotsarelis, Maksim V. Plikus

Last updated by author(s): Dec 18, 2018

Reporting Summary

Nature Research wishes to improve the reproducibility of the work that we publish. This form provides structure for consistency and transparency in reporting. For further information on Nature Research policies, see Authors & Referees and the Editorial Policy Checklist.

Statistics

For all statistical analyses, confirm that the following items are present in the figure legend, table legend, main text, or Methods section.

n/a	Confirmed	
☐	☒	The exact sample size (*n*) for each experimental group/condition, given as a discrete number and unit of measurement
☐	☒	A statement on whether measurements were taken from distinct samples or whether the same sample was measured repeatedly
☐	☒	The statistical test(s) used AND whether they are one- or two-sided<br>*Only common tests should be described solely by name; describe more complex techniques in the Methods section.*
☒	☐	A description of all covariates tested
☐	☒	A description of any assumptions or corrections, such as tests of normality and adjustment for multiple comparisons
☐	☒	A full description of the statistical parameters including central tendency (e.g. means) or other basic estimates (e.g. regression coefficient) AND variation (e.g. standard deviation) or associated estimates of uncertainty (e.g. confidence intervals)
☒	☐	For null hypothesis testing, the test statistic (e.g. *F*, *t*, *r*) with confidence intervals, effect sizes, degrees of freedom and *P* value noted<br>*Give P values as exact values whenever suitable.*
☐	☒	For Bayesian analysis, information on the choice of priors and Markov chain Monte Carlo settings
☒	☐	For hierarchical and complex designs, identification of the appropriate level for tests and full reporting of outcomes
☐	☒	Estimates of effect sizes (e.g. Cohen's *d*, Pearson's *r*), indicating how they were calculated

Our web collection on statistics for biologists contains articles on many of the points above.

Software and code

Policy information about availability of computer code

Data collection	Initial processing of 3'-end single-cell RNA-sequencing data was performed using the commercial Cell Ranger pipelines (10X Genomics, versions 2.1.0, as described in the Methods section of the manuscript). For 3'-end single-cell RNA-sequencing, transcripts were mapped to the mm10 reference genome (GRCm38.91) using Cell Ranger (version 2.1.0). For full length single-cell RNA-sequencing, demultiplexed, paired-end FASTQs were aligned to the mouse genome (mm10/gencode.Mv13) using Bowtie (version 1.0.0) and quantified using the RNA-seq by Expectation-Maximization algorithm (RSEM) (version 1.2.31), as described in the Methods section of the manuscript.
Data analysis	All downstream bioinformatic analysis was done in RStudio (version 3.4) and Matlab (version R2017b) software environment. Clustering of cells was performed using the Seurat R package (version 2.1, https://satijalab.org/seurat/install.html). Pseudotime analysis was performed using Monocle 2 (https://bioconductor.org/packages/release/bioc/html/monocle.html) and scEpath (version1, https://github.com/sqjin/scEpath). RNA velocity analysis was performed using velocyto (version 0.6, http://velocyto.org/). Quantile normalization was performed using SCnorm (version 1.2, https://github.com/rhondabacher/SCnorm) and batch correction was done using Combat from the sva R package (version 3.30.0, http://bioconductor.org/packages/release/bioc/html/sva.html).

For manuscripts utilizing custom algorithms or software that are central to the research but not yet described in published literature, software must be made available to editors/reviewers. We strongly encourage code deposition in a community repository (e.g. GitHub). See the Nature Research guidelines for submitting code & software for further information.

Data

Policy information about availability of data

All manuscripts must include a data availability statement. This statement should provide the following information, where applicable:

- Accession codes, unique identifiers, or web links for publicly available datasets
- A list of figures that have associated raw data
- A description of any restrictions on data availability

The authors declare that all supporting data are available within the article and its supplementary information files. All single-cell RNA-seq data have been deposited in the GEO database. 3'-end single-cell RNA-seq data is available under accession code GSE113854 (https://www.ncbi.nlm.nih.gov/geo/query/acc.cgi?

acc=GSE113854). Full length single-cell RNA-seq data is available under accession code GSE113605 (https://www.ncbi.nlm.nih.gov/geo/query/acc.cgi?acc=GSE113605).

Field-specific reporting

Please select the one below that is the best fit for your research. If you are not sure, read the appropriate sections before making your selection.

☒ Life sciences ☐ Behavioural & social sciences ☐ Ecological, evolutionary & environmental sciences

For a reference copy of the document with all sections, see nature.com/documents/nr-reporting-summary-flat.pdf

Life sciences study design

All studies must disclose on these points even when the disclosure is negative.

Sample size	No sample size calculations were performed for mouse experiments. Mouse wounds were pooled for single-cell RNA-seq experiments, which involved the analysis of many individual cells. For immunohistochemical analysis, we used 3 mice as biological replicates. For all bone marrow transplantation (BMT) experiments, 3 or more mice were used per experiment. For all Cre recombinase -based lineage tracing and gene deletion experiments, 9 or more mice were used per experiment.
Data exclusions	In 3'-end single-cell RNA-seq experiments, cells displaying more than 8,000 UMI/cell, 2,500 genes/cell, and more than 8% mitochondrial gene expression were excluded as low quality cells. Approximately 503 cells were excluded. For full length single-cell RNA-seq, cells with a total number of expressed genes > 11,000, a proportion of counts in mitochondrial genes > 15%, and genes expressed in < 3 cells were excluded as low quality cells. 56 cells were excluded from all replicates.
Replication	Key findings were reproduced throughout the manuscript. For example, immunohistochemical analysis recapitulated the expression profiles of fibroblast subtypes described in the initial 3'-end single-cell RNA-seq experiment. Key functional studies were also independently replicated. These include BMT and Cre recombinase-based lineage tracing assays. In single-cell RNA-seq experiments, individual sequenced cells were considered as replicates. In all in vivo mouse experiments, at least 3 mice were used per experiment. In adipocyte differentiation assays, technical replicates were used.
Randomization	Littermate mice were assigned into groups on the basis of genotype.
Blinding	Single-cell RNA-seq analyses were unbiased. All cells were analyzed using computational algorithms that were not biased to recognize any particular cell types. All mouse lineage tracing experiments involved identification of genetically labeled cells and did not require blinding. Adipocyte regeneration phenotype after Pparg deletion was quantified using blinded approach, by investigators who did not know the genotype.

Reporting for specific materials, systems and methods

We require information from authors about some types of materials, experimental systems and methods used in many studies. Here, indicate whether each material, system or method listed is relevant to your study. If you are not sure if a list item applies to your research, read the appropriate section before selecting a response.

Materials & experimental systems

n/a	Involved in the study	
☒	☐	Antibodies
☐	☒	Eukaryotic cell lines
☒	☐	Palaeontology
☐	☒	Animals and other organisms
☒	☐	Human research participants
☒	☐	Clinical data

Methods

n/a	Involved in the study	
☒	☐	ChIP-seq
☐	☒	Flow cytometry
☒	☐	MRI-based neuroimaging

Eukaryotic cell lines

Policy information about cell lines

Cell line source(s)	Primary mouse cells isolated from wounds on day 12 post-wounding and subsequently assayed in vitro, in adipocyte differentiation experiments.
Authentication	n/a
Mycoplasma contamination	n/a
Commonly misidentified lines (See ICLAC register)	n/a

Animals and other organisms

Policy information about studies involving animals; ARRIVE guidelines recommended for reporting animal research

Laboratory animals	The following mouse models were used in this study: C57BL/6J (JAX stock 000664), Retn-lacZ, Sm22-Cre (JAX stock 004746), Cd45-Cre, LysM-Cre (JAX stock 004781), Fabp4-Cre (JAX stock 005069), Pparγ-flox (JAX stock 004584), R26R (JAX stock 003474), tdTomato (JAX stock 007909), GFP (JAX stock 004353), RFP (JAX stock 006051), Rag1-/- (JAX stock 002216). All mice were housed under standard conditions and fed standard chow and water ad libitum. For wounding experiments, animals were anesthetized with isoflurane, hairs were clipped, skin site was disinfected and a single full thickness excisional wound was created on their dorsum using scissors (squared s = 1.5 cm). Both male and female mice between 6 to 8 weeks of age were used. Following wounding, all animals were housed individually. Wounds were let to heal by secondary intention. No wound dressing was applied. Animals were used as biological replicates.
Wild animals	This study did not involve wild animals.
Field-collected samples	This study did not involve animals collected from the field.
Ethics oversight	All animal experiments were carried out in accordance with the guidelines of the Institutional Animal Care and Use Committee of the University of Pennsylvania and University of California, Irvine.

Note that full information on the approval of the study protocol must also be provided in the manuscript.

Flow Cytometry

Plots

Confirm that:

☒ The axis labels state the marker and fluorochrome used (e.g. CD4-FITC).

☒ The axis scales are clearly visible. Include numbers along axes only for bottom left plot of group (a 'group' is an analysis of identical markers).

☒ All plots are contour plots with outliers or pseudocolor plots.

☒ A numerical value for number of cells or percentage (with statistics) is provided.

Methodology

Sample preparation	Bone marrow cells were flushed from the long bones with Dulbecco's Modified Eagle's Medium supplemented with 5% heat-inactivated calf serum (Gibco). To obtain a single cell suspension, cells were filtered through a 45 μm nylon screen. Cells were blocked with anti-rat and anti-mouse IgG (Sigma) for 15 minutes.
Instrument	BD FACS Aria II
Software	FlowJo software
Cell population abundance	The purity of sorted cells was >90% as tested by running post-sort samples on flow cytometer machine.
Gating strategy	For isolation of hematopoietic stem cells (HSCs), cells were stained with CD45 (APC-Cy7, BD Pharmingen), CD150 (APC, Biolegend), CD48 (Pacific Blue, Biolegend), SCA1(PE-Cy5.5, Invitrogen) and c-KIT (PE-Cy7, eBioscience), as well as PE-conjugated lineage markers including CD19, B220, CD3, CD4, CD8, GR-1, MAC-1, NK1.1, and TER119 (eBioscience). Cells were resuspended in DAPI to discriminate live from dead cells. HSCs were sorted by isolating Lineage-neg,SCA1+,c-KIT+,CD150+,CD48-neg cells using flow cytometer.

☒ Tick this box to confirm that a figure exemplifying the gating strategy is provided in the Supplementary Information.
